# Forty thousand kilometers under quantum protection

**DOI:** 10.1038/s41598-023-35579-6

**Published:** 2023-05-30

**Authors:** N. S. Kirsanov, V. A. Pastushenko, A. D. Kodukhov, M. V. Yarovikov, A. B. Sagingalieva, D. A. Kronberg, M. Pflitsch, V. M. Vinokur

**Affiliations:** Terra Quantum AG, St. Gallen, 9000 Switzerland

**Keywords:** Materials science, Physics

## Abstract

Quantum key distribution (QKD) is a revolutionary cryptography response to the rapidly growing cyberattacks threat posed by quantum computing. Yet, the roadblock limiting the vast expanse of secure quantum communication is the exponential decay of the transmitted quantum signal with the distance. Today’s quantum cryptography is trying to solve this problem by focusing on quantum repeaters. However, efficient and secure quantum repetition at sufficient distances is still far beyond modern technology. Here, we shift the paradigm and build the long-distance security of the QKD upon the quantum foundations of the Second Law of Thermodynamics and end-to-end physical oversight over the transmitted optical quantum states. Our approach enables us to realize quantum states’ repetition by optical amplifiers keeping states’ wave properties and phase coherence. The unprecedented secure distance range attainable through our approach opens the door for the development of scalable quantum-resistant communication networks of the future.

## Introduction

The quantum threat to secure communications makes top headlines and Niagara Falls of reviews and research explaining how quantum computers using, for example, Shor’s algorithm^1^, devalue the existing cryptographic schemes. Remarkably, the same advances in quantum physics that have created this quantum threat enable solutions for quantum security. Building upon quantum phenomena, novel quantum cryptography offers methods for unparalleled security, including quantum secure direct communication^2–7^, probabilistic one-time programs^8–10^, and quantum key distribution (QKD)^11–17^. The QKD, on which we focus in our present work, allows two parties to share a secret bit sequence for various applications. The existing QKD protocols are efficient over relatively short distances due to the fundamental Pirandola–Laurenza–Ottaviani–Banch (PLOB) bound^18^, which dictates that secret communication rates decrease exponentially with channel length. The simplest approach to resolve this issue is to use the trusted reproduction nodes along the transmission line^19–21^, which is a compromise to the overall security. The alternative solution is the utilization of quantum repeaters^22–37^ which eliminates the need for trust in the intermediate relay. However, since quantum repetition manipulates fragile entangled states, its implementation at a long scale remains beyond state-of-the-art technologies.

Here, contemplating the physical nature of the quantum states’ transmission, we lift the PLOB bound by using restrictions of quantum thermodynamics and the end-to-end physical control over losses in the optical quantum channel. We shift the quantum cryptography paradigm building on the same quantum considerations that provide the foundations of the Second Law of Thermodynamics. Our approach ensures signal repetition through optical amplification, presumes no trust at the intermediate channel points, and expands the secure transmission range to global distances. This paves the way for constructing scalable quantum communication networks of the future—a problem that has garnered significant interest in recent years^38–40^.

## General idea

Conventionally, the eavesdropper (Eve) is seen as capable of exploiting all the losses from the transmission channel, irrespective of their origin. This puts a strong restriction on the number of photons in the transmitted quantum states, which significantly complicates their repetition. However, upon close quantum mechanical examination, this presupposition appears unrealistic. In reality, the majority of losses in optical fibers occur due to the light scattering on the quenched disorder and are distributed homogeneously along the line (hereinafter, we will be referring to such losses as to natural losses). In a single mode silica fiber’s 1530–1565 nm wavelength window, the standard for modern telecommunications, these losses amount to approximately $4\times 10^{-5}$ of the passing signal’s intensity per meter.

We describe the information dynamics of the randomized signal transmitted over an optical channel. This consideration is carried out analogously to consideration of the Second Law of Thermodynamics, i.e., the dynamics of entropy, through the lens of the microscopic quantum mechanical laws^41–43^. Had the system been isolated, its entropy would not decrease, i.e., Eve would not be able to obtain any information. In the presence of natural losses, the system can no longer be regarded as isolated, and thus, the eavesdropper gets an opportunity to decrease the system’s entropy in analogy with the quantum Maxwell demon. However, in order to glean information from the scattering losses of relatively weak signals that we employ for our approach, Eve has to use quantum detection devices spanning an unfeasible length of optical fiber, see Supplementary Note 1. That is why one concludes that in this weak signal regime, Eve is unable to effectively collect and exploit natural losses.

Losses other than natural ones can, in turn, be physically controlled. We propose a technique of physical loss control (line tomography) implying that legitimate users detect local interventions by comparing the constantly updated tomogram of the line with the initial one, knowingly obtained in the absence of Eve. Line tomography involves sending the high-frequency test light pulses and analyzing their reflected (via the technique known as the time-domain reflectometry^44^) and the transmitted components. The coupling of photons to any eavesdropping system is impossible without modifying the fiber medium, which in turn inevitably changes the line tomogram. Unable to perform such radical interventions unnoticed, Eve is thus restricted to introducing small local leakages, which are precisely measured by the users. This implicates the possibility of employing the information-carrying light states containing the numbers of photons that are sufficient to repeat the states through optical amplification yet not enough to be easily eavesdropped on.

Utilizing a cascade of accessible optical amplifiers to counteract the degradation of signals over extensive distances, as opposed to the employment of quantum repeaters^22–37^, enables global transmission and high key distribution rates. It is important to note that these optical amplifiers should not be viewed as trusted nodes, as the integrity of the transmission scheme is maintained through end-to-end control by legitimate users, and there is no recourse to the form of classical data.

We showcase our approach via a prepare-and-measure QKD protocol utilizing non-orthogonal coherent photonic states ${|{\gamma _0}\rangle }$ and ${|{\gamma _1}\rangle }$ for encoding 0 and 1 bits. In the protocol’s framework, our approach means restricting the fraction of photons leaked to Eve, $r_\text {E}$, to ensure that the leaked states ${|{\sqrt{r_\text {E}}\gamma _{0}}\rangle }$ and ${|{\sqrt{r_\text {E}}\gamma _{1}}\rangle }$ sufficiently overlap, i.e., $\left| \left\langle \sqrt{r_\text {E}}\gamma _{0}\big |\sqrt{r_\text {E}}\gamma _{1}\right\rangle \right| \sim 1$ (these states become mixed if the transmission channel includes amplifiers, but for now we ignore this fact for the sake of simplicity). Eve cannot by any means—except by completely blocking part of the signal pulses^45^ but this is prevented by the line tomography—extract more information than the Holevo quantity $\chi _\text {E}$^46^ which tends to zero when $\left| \left\langle \sqrt{r_\text {E}}\gamma _{0}\big |\sqrt{r_\text {E}}\gamma _{1}\right\rangle \right| \rightarrow 1$. The users monitor the value of $r_\text {E}$ and adapt the parameters of ${|{\gamma _0}\rangle }$ and ${|{\gamma _1}\rangle }$ to ensure that the intercepted pulses are poorly distinguishable.

Thus, we overcome the PLOB bound by what can be called the “channel device-dependent” approach. This approach is no less physically justified as a traditional device-dependent scenario where the eavesdropper is assumed not to be able to substitute some of the equipment at the sender and receiver side. Hence, there are no compromises in security that allow us to increase the secret key distribution distance, only higher device dependence, with correct channel work ensured by tomography methods.

## Protocol description

We put forth an exemplary QKD protocol based on our physical control approach. Let the legitimate users, the sender, Alice, and the receiver, Bob, be connected via a classical authenticated communication channel and optical line serving as a quantum channel. The protocol is designed as follows:

**Initial preparation**

0. Alice and Bob carry out initial line tomography to determine the natural losses that Eve cannot exploit. At this and only this preliminary step, the legitimate users must be certain that Eve has no influence on the line. The users share the tomogram via the classical channel.

**Line tomography**

1. Alice and Bob perform the physical loss control over the line and, through comparison with the initial line tomogram, infer the fraction $r_\text {E}$ of the signal possibly seized and exploited by Eve. The users also localize the points of Eve’s intervention. To update the line tomogram, users exchange information via the classical channel. If the stolen fraction grows too large so that the evaluated legitimate users’ information advantage over Eve disappears—the analytical estimate for this advantage is provided below—the transmission is terminated.

**Transmission of quantum states**

2. Using a random number generator, Alice produces a bit sequence of the length *L*. Alice ciphers her bit sequence into a series of *L* coherent light pulses, which she sends to Bob. The bits 0 and 1 are encoded into coherent states ${|{\gamma _0}\rangle }$ and ${|{\gamma _1}\rangle }$, respectively. Their parameters are optimized based on the known fraction of the signal seized by Eve $r_\text {E}$ and Eve’s position in the line. The optimal parameters correspond to the maximum key distribution rate at given losses in the channel, the analytical relation for which is presented below. The optimal parameters are considered to be known to Alice and Bob and also to Eve.

3. The signals are amplified by the cascade of optical amplifiers installed along the optical line, possibly equidistantly. Bob receives the signals and measures them.

**Classical post-procession**

4. Alice and Bob perform the postselection, i.e., they discard the positions corresponding to corrupted measurement outcomes. The postselection criteria are defined by the set of parameters, which are optimally calculated by the users.
5. The users perform error correction. The procedure can be done with well-known classical methods, e.g., linear codes^47–49^, or with methods designed specifically for the QKD, such as the Cascade protocol^50–52^.
6. The users estimate Eve’s information obtained at the previous stages and perform the privacy amplification procedure to produce a shorter key (e.g., using the universal hashing method^53^) on which Eve has none or negligibly small information.

The steps from 1 to 6 directly constitute the process of key distribution, and the users must repeat them until satisfied with the total shared key length. It is important to note that the key is not generated solely by Alice in step 2; instead, it emerges through the collaborative simultaneous actions of both parties, with postprocessing playing a vital role in the process.

The particular way of encoding bits 0 and 1 into the parameters of coherent pulses ${|{\gamma _0}\rangle }$ and ${|{\gamma _1}\rangle }$ may vary. For illustrative purposes, we concentrate on the two simplest and straightforward schemes, viz. encoding bits into pulses with (a) different photon numbers, $|\gamma _0|^2 \ne |\gamma _1|^2$, and phase randomization^54–56^, and (b) same photon numbers, $|\gamma _0|^2 =|\gamma _1|^2$, and phases different by $\pi$. In both encoding schemes, Alice varies $|\gamma _0|^2$ and $|\gamma _1|^2$. More sophisticated encoding schemes, for instance, schemes leveraging the pulses’ shapes, make exploiting the natural scattering losses even more unsolvable. To complicate the problem further, the cable design may include an encapsulating layer of metal of heavily doped silica, transforming the scattering radiation into heat under the control of the users; see Supplementary Note 2 for details.

## Protocol security

Here, we delve into the security of the described protocol, by building upon the following:

1. Alice and Bob each generate random numbers that Eve cannot predict.
2. Other from the transmission channel—which is a fiber line with the embedded optical amplifiers—and the classical authenticated channel, users’ equipment is isolated from Eve.
3. Eve cannot effectively collect and exploit natural losses from the transmission channel. To eavesdrop on the signal, Eve must introduce new artificial local leakages. Eve can also use the local leakages on the original fiber discontinuities, such as bends or connections.
4. The transmission line between Alice and Bob is characterized by the initial line tomogram. All losses constituting deviations from the initial tomogram are attributed to Eve.
5. Eve is bound to the beam-splitting attack. She may seize some fraction of the signal at any point of the optical line. With that, she is unable to replace any section of the line with a channel of her own creation since this action is detectable by the line tomography.

Attacks that deviate from the beam-splitting attack necessitate a significant alteration of the line tomogram, in which case the protocol should be terminated; as such, we will not delve into them here. We will refer to the point of Eve’s intrusion into the line as the “beam splitter” and assume that any reflection back towards Alice from this point is insignificant. As our analysis will demonstrate, the protocol’s efficiency is contingent on Eve’s placement along the line. For the sake of simplicity, we will focus on the scenario in which Eve intercepts from a single point at the line. Indeed, with some overhead, the case where Eve intercepts from multiple points—stealing $r_\text {E}^{(i)}$ from the *i*-th local leakage—can be reduced to a situation where Eve is at the single worst location for the users among all identified interception points, and she steals the effective overall leakage

1 $$\begin{aligned} r_\text {E}=\sum _i r_\text {E}^{(i)}\prod _{0\le j<i}(1-r_\text {E}^{(j)})=1-\prod _{i}(1-r_\text {E}^{(i)}), \end{aligned}$$

where we defined $r_\text {E}^{(0)}=0$. This effective overall leakage is detectable by transmittometry, see “Physical loss control and amplification” for details. The detailed analysis of the multi-point interception and constructive interference will be the subject of our forthcoming publication.

To evaluate the protocol’s security, we describe the evolution of Alice’s, Bob’s, and Eve’s quantum systems and quantify the information available to different parties. We examine the case in which the beam splitter is placed immediately following one of the amplifiers, as this arrangement is most advantageous for Eve, but, with minimal adjustments, the same analysis can be applied to any arbitrary beam splitter’s position. We derive an analytical expression for the length of the final secure key $L_\text {f}$, which represents the users’ informational advantage over Eve given the fixed value of $r_\text {E}$ and the distance between Alice and Eve $D_{\text {AE}}$. This expression depends on the encoding and postselection parameters and should be maximized by the users to determine the parameters’ optimal values. The condition $L_{\text {f}}/L>0$ for the chosen parameters ensures successful secret key generation^57^.

At the beginning of the protocol, Alice encodes the logical bits into the coherent states with the different complex amplitudes, $0 \rightarrow {|{\gamma _0}\rangle },\,1 \rightarrow {|{\gamma _1}\rangle }$. In the photon number encoding scheme, the pulses are different in the average numbers of photons $|\gamma _0|^2$ and $|\gamma _1|^2$, while the phase of each pulse is random. The photon number measurement at Bob’s end is formalized in terms of the projective operators:

2a $$\begin{aligned} \hat{E}_0 = \sum \limits _{k=\mu -\theta _3}^{\mu -\theta _1} {|{k}\rangle } {\langle {k}|},\quad \hat{E}_1 = \sum \limits _{k=\mu +\theta _2}^{ \mu +\theta _4} {|{k}\rangle } {\langle {k}|},\quad \hat{E}_\text {fail} = \hat{\mathbbm {1}} - \hat{E}_0 - \hat{E}_1, \end{aligned}$$

where $\hat{E}_0$, $\hat{E}_1$ and $\hat{E}_\text {fail}$ correspond to 0, 1, and failed—meaning that this result should be later discarded—outcomes respectively, ${|{k}\rangle }$ is the Fock state of *k* photons, $\hat{\mathbbm {1}}$ is the identity operator, $\mu =\left( \left| \gamma _0\right| ^2+\left| \gamma _1\right| ^2\right) /2$, and $\theta _{1-4}$ are the postselection parameters tuned by Bob depending on the proportion of the stolen signal. The photon numbers between $\mu -\theta _1$ and $\mu +\theta _2$ are difficult to relate to 0 or 1, while numbers below $\mu -\theta _3$ and above $\mu +\theta _4$ are associated with the information corruption: as we show in Supplementary Note 4, optical amplification imposes correlations between pulses received by Bob and Eve, and extreme photon numbers at Bob’s end also constitute very distinguishable signals for Eve.

For the phase encoding (note that using this scheme requires that the optical fiber is phase-preserving), the pulses are characterized by the same average photon number, $|\gamma _0|^2=|\gamma _1|^2$, but by different, although fixed, phases. For instance, the relative phase can be $\pi$, $\gamma _0=-\gamma _1=\gamma \in \mathbb {R}$, and then, to distinguish the pulses, Bob should perform homodyne measurement of the quadrature $\hat{q}$ corresponding to the real axis in the phase space:

2b $$\begin{aligned} \hat{E}_0 = \int \limits ^{\theta _{\text {2}}^\prime }_{\theta _{\text {1}}^\prime } d q {|{q}\rangle }{\langle {q}|},\quad \hat{E}_1 = \int \limits ^{-\theta _{\text {1}}^\prime }_{-\theta _{\text {2}}^\prime } d q {|{q}\rangle }{\langle {q}|},\quad \hat{E}_\text {fail} = \hat{\mathbbm {1}} - \hat{E}_0 - \hat{E}_1, \end{aligned}$$

where ${|{q}\rangle }$ is the eigenstate of $\hat{q}$, and $\theta _{\text {1,2}}^\prime$ play the same role as $\theta _{1-4}$ in the photon number encoding case. With this scheme, we deal only with two postselection parameters because probability distributions of measurement results for two pulses are symmetric with respect to $q=0$.

For both encoding schemes, the operational values of $|\gamma _0|$, $|\gamma _1|$ and $\theta _{1-4}$ ($\theta _{\text {1,2}}^\prime$) are determined via maximizing the analytical expression for the predicted length of the final secure key $L_\text {f}$, which, in turn, depends on the proportion of the stolen signal $r_\text {E}$ and the distance between Alice and Eve $D_\text {AE}$. Correlations between the states at Eve’s and Bob’s disposal due to optical amplification drastically complicate the analytical description of states’ evolution necessary for obtaining the expression for $L_\text {f}$. We provide such a description in “Methods”, while here we write the final state of the combined quantum system of Alice’s random bit (A), the signal component seized by Eve (E), and Bob’s memory device storing the measurement outcome (B) after the legitimate users discard invalid bits, i.e., conditional to the successful measurement outcome:

3 $$\begin{aligned} \hat{\rho }^{\text {f}}_\text {ABE}{} & {} = \sum \limits _{b=0,1} \sum \limits _{a=0,1} \frac{1}{2p(\checkmark |a)} \int \!d^2 \alpha \,\, P(\alpha ,\sqrt{T_1}\gamma _a, G_1)\int \!d^2\beta \,\, {\langle {\beta }|}\hat{E}_b{|{\beta }\rangle }\nonumber \\{} & {} \quad \times P\left( \beta , \sqrt{(1-r_\text {E})T_2}\alpha ,G_2\right) {|{a}\rangle }{\langle {a}|}_\text {A} \otimes {|{b}\rangle }{\langle {b}|}_\text {B} \otimes {|{\sqrt{r_\text {E}}\alpha }\rangle }{\langle {\sqrt{r_\text {E}}\alpha }|}_\text {E}, \end{aligned}$$

where

4 $$\begin{aligned} P(\alpha ,\gamma ,G) = \frac{1}{\pi (G-1)} \exp \left( -\frac{|\alpha - \sqrt{G} \gamma |^2}{G-1}\right) , \end{aligned}$$

is the *P*-function describing amplification (see “Physical loss control and amplification” and Supplementary Note 3), and integration operations are performed over the complex plane, i.e., $d^2 \alpha \equiv d\text {Re}(\alpha )\,d\text {Im}(\alpha )$, $T_{1(2)}$ and $G_{1(2)}$ are, respectively, the transmission probability and amplification factor (the ratio of the output photon number to the input one of an amplification channel) of the effective loss and amplification channels equivalent to the cascade of amplifiers and losses before (after) Eve’s beam splitter (these values depend on the distances between Alice and Eve, $D_\text {AE}$, between Alice and Bob, $D_\text {AB}$, and between neighboring amplifiers, *d*, see Eqs. (58–60) in Supplementary Note 3), $p(\checkmark |a)$ is the probability of the successful measurement outcomes in the case that Alice sends bit $a=\{0,1\}$ (the explicit form is given by Eqs. (17) and (18) in “Methods”).

In the case of photon number encoding, Alice randomizes the phase of each pulse. As a result, neither Bob nor Eve would know the phase $\varphi$ of the incident pulse ${|{\gamma _a}\rangle }={|{|\gamma _a|e^{i \varphi }}\rangle }$ which effectively means that the final state of the combined system is described by $\hat{\rho }^{\text {f}}_\text {ABE}$ from Eq. (3) averaged over $\varphi$ (see Supplementary Note 4 for details):

5 $$\begin{aligned} \left\langle \hat{\rho }_\text {ABE}^{\,f}\right\rangle _\varphi{} & {} = \sum \limits _{b=0,1} \sum \limits _{a=0,1} \frac{1}{2p(\checkmark |a)} \frac{1}{2\pi } \int \limits _{0}^{2\pi }\!\!d\varphi \cdot \int \!\!d^2\alpha \,\, P(\alpha ,\sqrt{T_1}|\gamma _a|e^{i\varphi },G_1)\nonumber \\{} & {} \quad \times {|{a}\rangle } {\langle {a}|}_\text {A} \otimes {|{b}\rangle }{\langle {b}|}_{\text {B}}\otimes \,\,\left| \sqrt{r_\text {E}}\alpha \right\rangle \left\langle \sqrt{r_\text {E}}\alpha \right| _\text {E} \cdot \int \!\!d^2\beta \,\,P\left( \beta ,\sqrt{(1-r_\text {E})T_2} \alpha , G_2 \right) \langle \beta |\hat{E}_b|\beta \rangle . \end{aligned}$$

After the invalid bits are discarded, the information available to Eve about the bits kept by Alice (per bit) is given by

6 $$\begin{aligned} I(\text {A},\text {E}) = S(\text {A})-S(\text {A}|\text {E}), \end{aligned}$$

where $S(\text {X})=-\text {tr}\left[ \hat{\rho }_\text {X} \log _2 \hat{\rho }_\text {X}\right]$ is the quantum von Neumann entropy of system X (which is A, B, E, or their combinations, the corresponding density matrices are obtained from Eq. (3), or Eq. (5) if there is phase randomization, by taking partial traces), and $S(\text {Y}|\text {X})=S(\text {XY})-S(\text {X})$ is the conditional entropy. We calculate the upper bound of $I(\text {A},\text {E})$ differently in the cases of photon number and phase encoding. In the first case, we use the Holevo bound^46^, see Supplementary Note 4. In the second case, we also rely on the concavity of relative entropy, see Supplementary Note 5.

**Figure 1:**
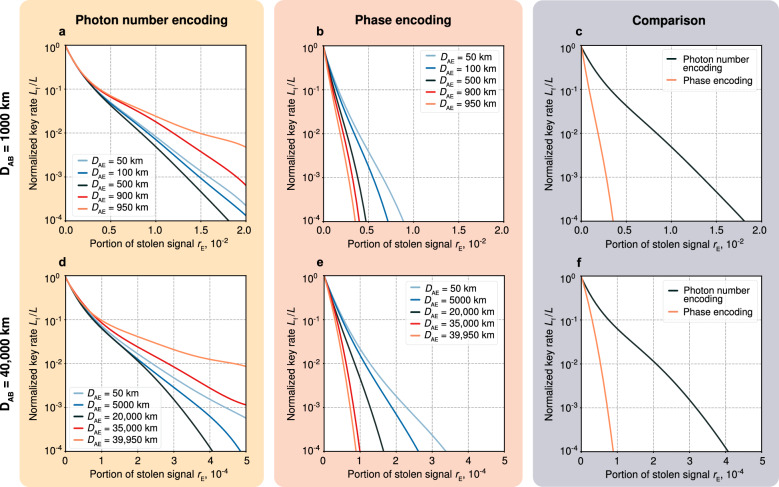
Numerical simulations of the protocol for different parameters and encoding schemes. (**a**) The normalized key rate $L_\text {f}/L$ as function of the proportion of stolen signal $r_\text {E}$ for the photon number encoding and $D_\text {AB}=1000\,\text {km}$. (**b**) The same for the phase encoding. (**c**) Comparison of the photon number and phase encoding schemes for $D_\text {AB}=1000\,\text {km}$. (**d**) $L_\text {f}/L(r_\text {E})$ for the photon number encoding and $D_\text {AB}=40{,}000\,\text {km}$. (**e**) The same for the phase encoding. (**f**) Comparison for the distance $D_\text {AB}=40{,}000\,\text {km}$. In all plots, the distance between neighboring amplifiers $d=50$ km. Different curves in each plot correspond to varying distances between Alice and Eve, $D_\text {AE}$. The dependence of $L_\text {f}/L$ on $D_\text {AE}$ is due to the fact that the amount of eavesdropped information is affected by correlations and noise imposed by optical amplifiers. The comparative plots (**c,f**) of two encoding schemes imply the respective worst conditions (with Eve positioned in her best way). In each point of every plot, the protocol’s parameters—i.e., the photon numbers $\left| \gamma _{0,1}\right| ^2$ and postselection parameters $\theta _{1-4}$ (or $\theta _{\text {1,2}}^\prime$)—are numerically optimized for the fixed values of $D_{\text {AB}}$, $D_{\text {AE}}$ and $r_\text {E}$ with respect to $L_\text {f}/L$. Depending on $r_\text {E}$, the optimal photon numbers $|\gamma _0|^2$ and $|\gamma _1|^2$ vary from $0.8\times 10^4$ to $3.0\times 10^4$ photons in (**a**), from $0.4\times 10^3$ to $7.2\times 10^3$ photons in (**b**), from $2.0\times 10^5$ to $3.0\times 10^5$ photons in (**d**), and from $0.4\times 10^5$ to $3.0\times 10^5$ photons in (**e**).

By performing the error correction procedure, the legitimate users establish a shared bit sequence (raw key) at the price of disclosing an additional error syndrome of the length $f\cdot S(\text {A}|\text {B})$, where $f \ge 1$ depends on the particular error correction code. As we do not intend to address any specific error correction method, we put $f=1$ corresponding to Shannon’s limit. After the procedure, Eve’s information becomes $\tilde{I}(\text {A},\text {E}) = I(\text {A},\text {E})+S(\text {A}|\text {B})$. To eradicate Eve’s information about the raw key, Alice and Bob perform the privacy amplification procedure tailored precisely for the estimated information leakage due to the local line losses and error correction, see “Methods”. The length of the final key is

7 $$\begin{aligned} L_\text {f}= p_\checkmark L\cdot \left( S(\text {A}) - \tilde{I}(\text {A},\text {E})\right) = p_\checkmark L\cdot \left( S(\text {A}) - S(\text {A}|\text {B})- I(\text {A},\text {E})\right) , \end{aligned}$$

where *L* is the number of originally generated random bits, and $p_\checkmark =\frac{1}{2}\sum _{a,b=0,1} p(b|a)$ is the proportion of bits that are not discarded at the postselection stage.

Taking *L* and $L_\text {f}$ as the numbers of bits per unit of time, Eq. (7)—the explicit form of which can be obtained using Eqs. (2a or 2b), (2 or 4), and Eqs. (17, 18) from “Methods”—gives us the key distribution rate (or key rate for short) as a function of $r_\text {E}$, $|\gamma _0|$, $|\gamma _1|$ and $\theta _{1-4}$ (or $\theta _{\text {1,2}}^\prime$). Implicitly, the equation also includes the distance between two neighboring amplifiers *d* and the distances between Alice and Bob, $D_{\text {AB}}$, and Alice and Eve, $D_{\text {AE}}$. As we specified above, the users obtain the optimal values of $|\gamma _0|$, $|\gamma _1|$ and $\theta _{1-4}$ (or $\theta _{\text {1,2}}^\prime$) by maximizing this analytic formula for measured values of $r_\text {E}$ and $D_{\text {AE}}$. To be able to distribute secret keys, the users have to possess an information advantage over Eve^57^, which in our case is indicated by the positivity of the calculated $L_\text {f}/L$. If the evaluated $L_\text {f}/L$ is not positive, the protocol should be terminated.

## Numerical simulations

Figure 1 displays the results of our numerical simulations. We plot the optimal normalized key rate $L_\text {f}/L$ as a function of the proportion $r_\text {E}$ for two different transmission distances: (a, b, c) $D_{\text {AB}}=1000$ km, (d–f) $D_{\text {AB}}=40{,}000$ km. The distance between the neighboring amplifiers $d=50$ km.

Plots  a, d relate to the photon number encoding with different curves corresponding to different values of $D_{\text {AE}}$. We observe that the worst normalized key rate $L_f/L$ occurs when Eve is close to the middle of the transmission line. This is explained by the side effects of signal amplification: the closer Eve is to Bob, the more Eve’s part of the signal is correlated with Bob’s, yet, the noisier it becomes (see Supplementary Note 4). With such a trade-off, Eve gets the largest amount of information, standing somewhere in the vicinity of the line’s midpoint. However, in the phase encoding case, shown in b, e panels, correlations outweigh noise even when Eve is close to Bob, resulting in a lower key rate for larger $D_{\text {AB}}$. Plots c, f show the protocol’s performance under both encoding schemes in the respective worst-case scenarios: Eve’s position is such that the key rate is the lowest. Therefore, as we see from the plots, the photon number encoding scheme appears to be more efficient.

As we show in “Methods”, see Supplementary Note 3 for technical details, the minimal detectable leakage for a long line with *M* equidistant amplifiers is $r_\text {E}^\text {min}\sim \sqrt{MG/n}$, where *G* is the amplification factor of a single amplifier, and *n* is the number of photons in a test pulse. With $d=50$ km, $G=10$, and $n=10^{14}$, we get $r_\text {E}^\text {min}\sim 10^{-6}$ and $r_\text {E}^\text {min}\sim 10^{-5}$ for the 1000 km ($M=20$) and 40,000 km ($M=800$) lines, respectively. Close to the loss control precision limit, both encoding schemes allow for high key rates. For the photon number encoding, the maximum $L_\text {f}/L$ is 0.99 for 1000 km and 0.57 for 40,000 km. For the phase encoding, the values are 0.98 and 0.27, respectively.

Within the selected ranges of $r_\text {E}$, which are above the minimum detectable leakage, and, therefore, such losses are resolvable by the physical loss control, and for the photon number encoding we have $L_\text {f}/L\gtrsim 10^{-4}$. Correspondingly, if the initial random number generation rate $L=1$ Gbit/s, then for 1000 km we have $0.99\,\text {Gbit/s}\gtrsim L_\text {f}\gtrsim 100\,$Kbit/s and for 40,000 km we have $0.57\,\text {Gbit/s}\gtrsim L_\text {f}\gtrsim 100\,$Kbit/s. In comparison, the asymptotic behavior of the normalized key rate provided by PLOB at a 1000 km distance is limited to values around $10^{-9}$, or 1 bit/s for $L=1$ Gbit/s, which is several orders of magnitude lower than the rates achievable with our method. To the best of our knowledge, there have been no previously reported QKD protocols that cover tens of thousands of kilometers without using trusted nodes. Furthermore, state-of-the-art Twin-Field QKD realizations^40,58,59^ offer key rates that do not exceed a few bits per second at distances comparable to 1000 km. At the same time, the QKD realizations featuring high secret key rates of the order of 1 Kbit/s span relatively short communication distances of a couple of hundred kilometers^60–62^.

## Physical loss control and amplification

Now we outline possible implementations of the basic technological components of the protocol, the physical loss control, and the signal repetition by optical amplifiers. The physical loss control methods are based on analyzing scattered components of the high-energy test pulses sent along the fiber. The optical time-domain reflectometry comprises the injection of test pulses into the fiber and subsequent measurement of the temporal sequence of their back-scattered components. The response delay defines the distance to a particular scattering point, while its magnitude reflects the respective losses. Moreover, characteristic features of the response allow for determining the nature of the detected line discontinuity, see the exemplary experimental reflectogram in Fig. 4 in “Methods”.

**Figure 2:**
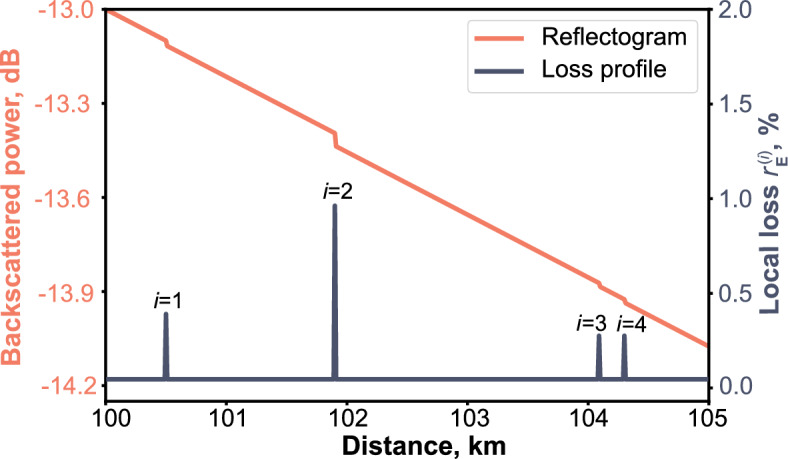
Exemplary reflectogram and loss profile. The loss profile, which displays the magnitude $r_\text {E}^{(i)}$ of the *i*-th local leakage and its position, is derived from the reflectogram using $\ell _1$-filtering.

As illustrated in Fig. 2, a log-linear reflectogram features a sequence of linear drops and steep drops (upper trace) corresponding to different discontinuities. To construct a loss profile using this piecewise linear graph, one can employ the $\ell _1$-filtering technique^63^, which is commonly used in the processing of reflectometry data^64,65^. This approach involves fitting the graph with a sum of a single linear decreasing function and a series of weighted step-like functions by minimizing the objective function based on the $\ell _1$ norm. The *i*-th step-like function’s drop (discrete derivative) and its position reveal the corresponding local leak magnitude $r_\text {E}^{(i)}$ and its respective location (lower trace). In turn, the linear decreasing component defines the homogeneously spread natural scattering losses.

Note, that to obtain an accurate reflectogram one has to make averaging over multiple test runs during which a test pulse travels to the end of the fiber and all its reflections return back. Accumulating sufficient statistics may, in reality, take a few seconds. To resolve this problem and to ensure high operational control speed, we develop the component of tomography which we call transmittometry: Alice sends test pulses comprising a large number of photons to Bob, they cross-check the sent and received photon numbers and obtain the proportion of the transmitted photons $(1-r_0)(1-r_\text {E})$. Knowing the natural losses baseline $r_0$, which is determined during the protocol’s initial preparation, they infer the the effective overall leakage $r_\text {E}$ as given by Eq. (1). In the spirit of the lock-in method^66,67^, the power of a test pulse can be modulated at high frequency so that the pulse contains a large number of periods. By comparing the input and output spectral power peaks at the modulation frequency, which can be determined through the Fourier transform of the time-dependent transmitted and received powers, the users can deduce the proportion of losses. Modulating the power helps to suppress the unwanted contribution of classical noise that may be present in the system. As the modulation frequency increases, the corresponding value of the noise spectral density tends to decrease, raising the transmittometry precision. Unlike reflectometry, transmittometry does not enable the users to localize and identify individual leakages but immediately updates the estimate of the effective overall leakage. Thus, the two control methods complement each other, the users are constantly aware of the magnitude of leakages and can localize them after accumulating sufficient reflectometry statistics.

To discriminate between the intrinsic and artificial line losses, the legitimate users prerecord the initial undisturbed line tomogram, including the reflectogram and the total proportion of losses in the line, and use this tomogram as a reference. The fiber material, silica, has an amorphous nonreproducible structure, making its reflectogram a physically unclonable function^68^. With that, the fiber core can be slightly doped, with, e.g., Al, P, N, or Ge, to tune its tomography results and achieve the optimal parameters such as dispersion. The most general eavesdropping attack implies a unitary transformation of the state of the combined system comprising the propagating signal and some ancillary eavesdropping system. However, coupling of photons to devices outside the line requires making significant alterations to the fiber medium, which would inevitably change the reflectogram and hence will be detected. Quantum cryptography also addresses attacks exercising the partial blocking of the signal and the subsequent unauthorized substitution of the blocked part. Any intervention like that would inevitably and permanently (even if Eve at some point decided to disconnect from the line) affect the tomogram of the transmission line and hence will be detected by the legitimate users.

The key distribution itself should go in parallel with accumulating the reflectometry statistics. If, at some point, the reflectogram shows an intrusion into the line, the users should respond with the appropriate postprocessing of the bits distributed during the formation of the reflectogram. This may possibly come down to discarding the whole bit sequence. Ideally, the physical loss control should be conducted permanently and should not halt even during the pauses in the key distribution. Taking the transmittometry test pulses’ duration of the order of 1 ns makes any real-time mechanical intrusion into the line immediately detectable.

**Figure 3:**
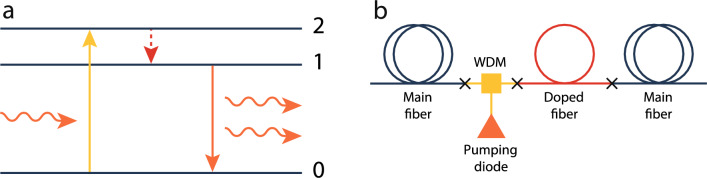
Optical amplification. (**a**) Energy diagram of the light amplification in the erbium-doped fiber section. Pumping radiation excites erbium ions from the ground state 0 into the 2nd energy level. Shortly after, ions drop to the metastable level 1. The incident photons stimulate the transition $1\rightarrow 0$ which results in the coherently synchronized radiation of additional photons at the same wavelength. (**b**) Schematics of the proposed bidirectional optical amplifier. The doped fiber section is embedded into the main fiber line and linked to the pumping diode through the WDM.

The principal task of the physical loss control is to ensure that Eve does not get enough photons to obtain the informational advantage over Bob. With that, signal pulses and test pulses still carry large numbers of photons, making it possible to repeat them via optical amplification. The repeater can particularly be arranged as a doped fiber section embedded into the main line and pumped to produce amplification gain in the primary mode. In telecommunications, the most common dopant is erbium. Pumped at the wavelength of 980 nm, the erbium-doped fiber generates the gain at around 1550 nm which fits into the transmission window of the silica-based fiber. Upon absorption of the pumping radiation, erbium ions transit from the ground state (state 0) to a short-lived state (state 2). From there, they non-radiatively relax to a metastable state (state 1), as illustrated in Fig. 3a. As the signal photonic mode passes through the inverted atomic medium, it stimulates the transition from state 1 to state 0, resulting in a coherently synchronized photon emission. The resulting signal amplification magnitude depends on the erbium ion concentration, the length of the doped fiber segment (active fiber), and the power of the pumping radiation. The interaction between the propagating signal photonic mode (with the annihilation operator $\hat{a}$) and the inverted atoms is determined by the Hamiltonian in the rotating wave approximation

8 $$\begin{aligned} \hat{H}_{\text {int}} = i\kappa \sum \limits _{n=1}^{N}\left( \hat{a}^\dag \otimes {|{0}\rangle }{\langle {1}|}_{n} - \hat{a}\otimes {|{1}\rangle }{\langle {0}|}_{n} \right) , \end{aligned}$$

where atoms are indexed by *n*, $N\gg 1$ is the total number of atoms in the medium, $\kappa$ is the interaction constant, and states ${|{0}\rangle }_n$ and ${|{1}\rangle }_n$ represent respectively states 0 and 1 of the *n*-th ion. Since the time of relaxation from state 2 to 1 is very small (20 $\upmu$s against 10 ms for the relaxation from 1 to 0), we take that an ion is effectively a two level system. The Holstein–Primakoff transformation maps the active medium’s state with *m* atoms in state 0 and $N-m$ in state 1 to a Fock state ${|{m}\rangle }_b$. This state corresponds to *m* excitations in the auxiliary bosonic mode associated with the annihilation operator $\hat{b}$. With that, we get the following equation

9 $$\begin{aligned} \hat{H}_{\text {int}} = i\kappa \sqrt{N}\left( \hat{a}^{\dagger }\hat{b}^{\dagger } - \hat{a}\hat{b} \right) . \end{aligned}$$

The evolution operator describing the signal mode propagation is given by

10 $$\begin{aligned} \hat{U}_g = e^{-i\hat{H}_{\text {int}} t/\hbar }=e^{g (\hat{a}^\dag \hat{b}^\dag - \hat{a} \, \hat{b})}, \end{aligned}$$

where $g = \kappa \sqrt{N}t/\hbar$, and *t* is the effective time of interaction between the signal mode and active medium. Amplification is described by a quantum channel acting on the signal’s density matrix $\hat{\rho }$

11 $$\begin{aligned} \text {Amp}_{G=\cosh ^2(g)}[\hat{\rho }]=\text {tr}_b \left[ \hat{U}_g\, \hat{\rho } \otimes {|{0}\rangle } {\langle {0}|}_b\, \hat{U}_g^\dag \right] , \end{aligned}$$

where the partial trace is taken over the states of the auxiliary mode. Within the *P*-function formalism, Eq. (11) translates into Eq. (4). Additional details can be found in Supplementary Note 3.

Usually, doped fiber amplifiers utilize optical isolators, which allow the light to pass only in one direction. This minimizes the risk of multiple reflections inside the doped fiber section. In our protocol, however, the optical isolators would block the reflected light hindering the end-to-end time-domain reflectometry. Besides, the amplifiers typically include tap couplers diverting about $1\%$ of the radiation into the photodetectors to monitor the amplifiers’ operation, and this fraction can possibly be seized by the eavesdropper. We hence opt out of both the optical isolators and tap couplers and utilize the design of the bidirectional optical amplifier^69–71^. The amplifier’s sketch is displayed in Fig. 3b. The fiber core is connected to the wavelength-division multiplexing (WDM) system. The WDM system is a beam splitter-like device for guiding the radiation of the different wavelengths into a single optical fiber. In our case, it is intended to feed the doped fiber section with the pumping radiation necessary to excite the active fiber’s dopant atoms. Correspondingly, the WDM is connected to the active fiber and the pumping diode. Finally, active fiber is connected to the main fiber line. Provided that the neighboring amplifiers are separated enough, they are not subject to significant cross-talk.

Our preliminary experiments reveal how the 1000 km-long line with the standard telecom distance $d=50$ km between amplifiers can be made stable with the very restricted signal noise in the line, even in the absence of optical isolators. The stability and scalability of our QKD scheme are supported by the remarkable precision of the loss control observed in the experiments. This control precision is accomplished through the use of high-resolution reflectometry, complemented by optical amplifiers to extend its range, and lock-in based transmittometry. With the ability to capture leakages above the control resolution, the users maintain an information advantage over potential adversaries attempting to exploit these leakages, as confirmed by analytical calculations. Coupled with the experimentally observed low bit error rates, our QKD system ensures high rates of secure key generation. We find that the signal wavelength of 1530 nm—corresponding to the peak of the amplification factor spectrum of the erbium-doped fiber amplifier—is more preferable than the standard 1550 nm wavelength. Fixing $G=1/T$ for 1550 nm means greater amplification for the noise in the modes near 1530 nm, which, in turn, may disrupt the stability of the amplifiers’ operation, possibly turning them into lasers. But this is not the case if 1530 nm is already the target wavelength itself.

A possible eavesdropping attack on the amplifier may consist of increasing the pumping power and stealing the surplus of the amplified radiation. Of course, hooking up to the line would change its tomogram and thus will be detected. Nevertheless, the following constructive feature of the amplifier will serve as an additional element of protection. The doped fiber section will contain the near minimum number of dopant ions necessary to amplify the signal with the target amplification factor. Let the operational pumping power $P_\text {p}$ match the target amplification factor *G*. With the increase of the pumping power, the relative population inversion asymptotically approaches unity, which corresponds to the amplification factor $G+\delta G$. The fraction of signal that Eve can possibly steal by inflating the pumping power is limited by $\delta G/G$, which is small, if at $P_\text {p}$ almost all of the ions are already excited. The number of ions and the value of $P_\text {p}$ should be such that the maximum achievable Eve’s makeweight, summed over all amplifiers installed into the line, is smaller than the minimum detectable leakage.

## Discussion and conclusions

Quantum cryptography typically assumes channel device independence, suggesting that an eavesdropper can fully exploit all leakages from the quantum channel. This assumption constrains key rates to the PLOB bound^18^, where the maximum rate scales as $-\log _2(1 - T)$ bits per channel with the transmittivity *T*. Consequently, key rates become impractically small over long distances.

By examining the physical quantum principles governing signal transmission and implementing physical loss control, we shifted this paradigm. In our approach, legitimate users can determine the fraction of losses accessible to an eavesdropper and ensure it contains insufficient information. While Eve struggles to discriminate between weak quantum states, Bob receives signals with relatively large numbers of photons, granting him a significant information advantage. Our approach employs end-to-end line tomography and leverages the impossibility of exploiting natural losses. As a result, the PLOB constraint is lifted, significantly extending the practical implementation of QKD over long distances without sacrificing security—notably, without relying on trusted nodes. Optical amplifiers, unlike trusted nodes, do not convert quantum information into classical form and are directly controlled by users through end-to-end control.

In this study, we analyzed a physical model restricting Eve to the possibility of local leakage exploitation, demonstrating the security of loss control-based QKD under long-distance transmission and high signal intensity conditions. Our forthcoming mathematical research will explore alternative physical models involving more complex actions by Eve and will provide further security proofs, particularly dealing with the finite key length and security parameter^72^.

The proposed approach maintains the fundamental advantage of the QKD, everlasting security^72–75^, ensuring that distributed keys will remain secure even against future technologies or attacks that may be developed. In the forthcoming publication, we will address the experimental realization of the QKD based on our approach for the transmission distance over 1000 km.

## Methods

### Combined quantum state evolution under beam splitting attack

Here we provide the description of states’ evolution in the case of the beam splitting attack. We will use that (a) an amplifier transforms pure coherent state into a mixture of the coherent states,

12 $$\begin{aligned} {|{\gamma }\rangle }\rightarrow \int d^2\alpha \,P(\alpha ,\gamma ,G) {|{\alpha }\rangle }{\langle {\alpha }|}, \end{aligned}$$

where $P(\alpha ,\gamma ,G)$ is given by Eq. (4) and integration is performed over the complex plane with $d^2 \alpha \equiv d\text {Re}(\alpha )\,d\text {Im}(\alpha )$; and that (b) formally, a sequence of losses and amplifications can be reduced to a single pair of the loss and amplification quantum channels—see Supplementary Note 3 for details.

The initial density matrix of Alice’s random bit (A) and the corresponding signal (S) is given by

13 $$\begin{aligned} \hat{\rho }^\text {i}_\text {AS}= \frac{1}{2}{|{0}\rangle }{\langle {0}|}_\text {A} \otimes {|{\gamma _0}\rangle }{\langle {\gamma _0}|}_\text {S} + \frac{1}{2}{|{1}\rangle }{\langle {1}|}_\text {A} \otimes {|{\gamma _1}\rangle }{\langle {\gamma _1}|}_\text {S}. \end{aligned}$$

Just before the signal passes the beam splitter, the state of the AS system is

14 $$\begin{aligned} \hat{\rho }^{\rightarrow \Box }_\text {AS} = \frac{1}{2} \sum \limits _{a=0,1} {|{a}\rangle } {\langle {a}|}_\text {A} \otimes \int d^2 \alpha \cdot P(\alpha ,\sqrt{T_1} \gamma _a,G_1) \cdot {|{\alpha }\rangle } {\langle {\alpha }|}_{\text {S}}, \end{aligned}$$

where we use Eq. (12) to describe the state of sequentially attenuated and amplified signal, and $T_1$ and $G_1$ are, respectively, the transmission probability and amplification factor of the effective loss and amplification channels that are equivalent to the sequence of amplifications and losses prior to the beam splitter, see Supplementary Note 3, particularly Eq. (58). Just after the signal passes the beam splitter, the state of the joint system of Alice’s random bit, the signal travelling to Bob and the signal component seized by Eve (E) is described by

15 $$\begin{aligned} \hat{\rho }^{\Box \rightarrow }_\text {ASE} = \frac{1}{2} \sum \limits _{a=0,1} {|{a}\rangle } {\langle {a}|}_\text {A} \otimes \int d^2 \alpha \cdot P(\alpha ,\sqrt{T_1} \gamma _a,G_1) \times {|{\sqrt{1-r_\text {E}} \alpha }\rangle } {\langle {\sqrt{1-r_\text {E}} \alpha }|}_\text {S} \otimes {|{\sqrt{r_\text {E}} \alpha }\rangle } {\langle {\sqrt{r_\text {E}} \alpha }|}_\text {E}, \end{aligned}$$

where $r_\text {E}$ is the fraction of signal stolen by Eve. After the signal passes the second series of losses and amplifiers and right before it is measured by Bob, the state of the joint system is

16 $$\begin{aligned} \hat{\rho }^{\rightarrow \text {Bob}}_\text {ASE}{} & {} = \frac{1}{2} \sum \limits _{a=0,1} {|{a}\rangle } {\langle {a}|}_\text {A} \otimes \int d^2 \alpha \cdot P(\alpha ,\sqrt{T_1} \gamma _a,G_1) \nonumber \\{} & {} \quad \times \left( \int d^2 \beta \cdot P\left( \beta ,\sqrt{(1-r_\text {E})T_2} \alpha , G_2 \right) \cdot {|{\beta }\rangle } {\langle {\beta }|}_\text {S} \right) \otimes {|{\sqrt{r_\text {E}} \alpha }\rangle } {\langle {\sqrt{r_\text {E}} \alpha }|}_\text {E}, \end{aligned}$$

where we again utilize Eq. (12) to describe the evolved signal state, and $T_2$ and $G_2$ are the effective transmission probability and amplification factor of the region between the beam splitter and Bob, see Eq. (60) in Supplementary Note 3. Bob receives the signal state, measures it and, together with Alice, discards the bits corresponding to the failed measurement results. The probability that Bob’s measurement outcome is $b=\{0,\,1\}$ given that Alice’s sent bit is $a=\{0,\,1\}$ can be written as

17 $$\begin{aligned} p(b|a)=\text {tr}_\text {ASE}\left[ {\left( 2\cdot {|{a}\rangle }{\langle {a}|}_\text {A} \otimes \hat{E}_b\otimes \hat{\mathbbm {1}}_\text {E}\right) \hat{\rho }^{\rightarrow \text {Bob}}_\text {ASE}}\right] , \end{aligned}$$

where $\hat{E}_b$ is given by Eq. (2a) or (2b) depending on the encoding scheme, and $\text {tr}_\text {ASE}[\dots ]$ is the trace over the ASE system. The probability that Bob performs a successful measurement if Alice sends bit *a* is

18 $$\begin{aligned} p(\checkmark |a)=p(0|a)+p(1|a). \end{aligned}$$

Equation (2) follows from Eq. (16) after applying measurement operators to the signal subsystem, discarding sum components associated with the failed outcomes, and renormalizing.

### Privacy amplification

Privacy amplification can be realized through applying the universal hashing method^53^, which requires the users to initially agree on the family $\mathcal {H}$ of hash functions. At the privacy amplification stage^50,76,77^, they randomly select such a function $h:\{0,1\}^{p_\checkmark L}\rightarrow \{0,1\}^{L_\text {f}}\in \mathcal {H}$ that maps the raw key of length $p_\checkmark L$ to the final key of length $L_\text {f}$. If Eve is estimated to know $p_\checkmark L\cdot \tilde{I}(\text {A},\text {E})$ bits of the raw key, the letter must be taken in accord with Eq. (7). Family $\mathcal {H}$ can, for example, span Toeplitz matrices^78^: a random binary Toeplitz matrix $\hat{T}$ with $p_\checkmark L$ rows and $L_\text {f}$ columns translates the binary vector representation of the raw key $\textbf{v}$ into the vector $\textbf{k}$ representing the final key, $\textbf{k} = \hat{T}\cdot \textbf{v}$.

**Figure 4:**
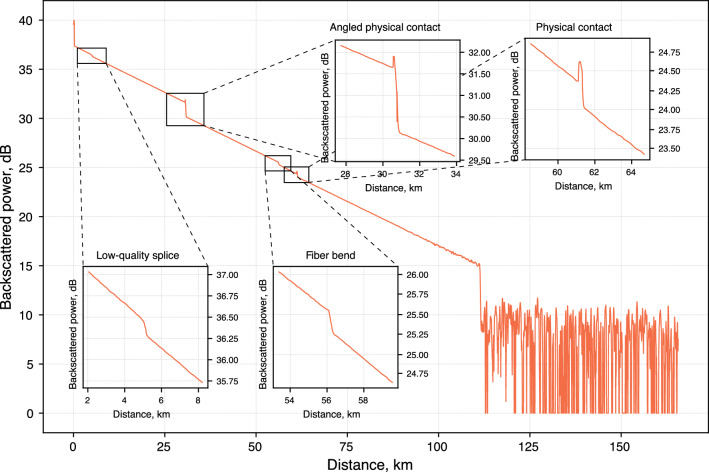
An exemplary plot obtained with the optical time-domain reflectometer. The device sends the high-intensity test pulses into the fiber and registers its reflections providing the dependence of the backscattered power on the distance to the scattering point defined by the time of the signal’s return.

### Physical loss control precision

Assume that Bob is equipped with an optical filter with a very narrow wavelength band which blocks noise from the secondary light modes (for details on this additional noise, see Supplementary Note 3). Assume also that all amplifiers are positioned equidistantly, each having amplification factor $G=1/T$ with *T* being the transmission probability of the line section between two neighboring amplifiers.

Approaching an amplifier, the test pulse comprising *n* photons is attenuated down to *Tn* photons. The amplifier restores the number of photons back to *n* but adds noise. The photons in the pulse follow the Poisson statistics; thus, the photon noise just before the amplifier can be taken as a square root of the number of photons in the input signal $\sqrt{Tn}$. The noise is amplified by factor *G* as well, so after a single amplifier the noise is $G \sqrt{T n}$. Coming through a sequence of *M* amplifiers which add fluctuations independently, the total noise raises by the factor $\sqrt{M}$. The noise at Bob’s end is thus $\delta n_\text {B}\simeq G \sqrt{MT n}=\sqrt{GM n}$. The minimum detectable leakage can be calculated as $r_\text {E}^\text {min}\sim \delta n_\text {B}/n=\sqrt{MG/n}$. Our qualitative estimates match with the detailed calculations in Supplementary Note 3.

### Experimentally obtained reflectogram

Figure 4 displays an example of an experimentally obtained reflectogram. Every particular type of fiber discontinuity, whether it is physical contact, bending, or splice, can be identified by its own unique reflectographic pattern, as demonstrated in the inset plots. The appearance of peaks signifies the excessive scattering which happens, for instance, at the physical connectors where the signal undergoes the Fresnel reflection. The right noisy tail of the main plot corresponds to the end of the backscattered signal. The measurements are carried out with a 2 $\upmu$s 1550 nm pulse laser with a power up to 40 mW. The experimental data is averaged over 16,000 measurements.

## Supplementary Information

Supplementary Information.
