# Supplementary material for: Forty thousand kilometers under quantum protection
Source: Sci Rep. 2023 May 30;13:8756. doi: 10.1038/s41598-023-35579-6 (PMC10229600; doi:10.1038/s41598-023-35579-6)
Supplement: Supplementary file 1 — Supplementary Information. [file 41598_2023_35579_MOESM1_ESM.pdf]

Supplementary Information: Forty Thousand Kilometers Under Quantum Protection

N. S. Kirsanov, V. A. Pastushenko, A. D. Kodukhov, M. V. Yarovikov, A. B. Sagingalieva, D. A. Kronberg, M. Pflitsch, and V. M. Vinokur

Terra Quantum AG

NOTE 1. Natural losses

In this note, we discuss the unfeasibility of eavesdropping on the natural losses occurring due to the scattering of photons in optical fiber. Here we explore quantum considerations analogous to those that serve to derive the Second Law of Thermodynamics [79–81].

In quantum cryptography, the thermodynamic considerations should focus on the collection of optical states traveling through a lossy fiber. In the standard telecommunication scenario, with the signal power of around 10 mW and a pulse duration of 1 ns, each light pulse contains about 10^8 photons, with staggering leaks of about 10^3 photons per meter that can be measured outside of the fiber. This scenario implies that the system is clearly not isolated, and, through the measurement of leaked photons, Eve can decrease the system's entropy—a process which, in accordance with Shannon's definition of entropy, is equivalent to extracting information.

On the other hand, in a relatively weak signal region of our choice with 10^4 to 10^5 photons or less (which is still enough for the optical amplification), the system can be considered quasi-isolated since only a small number of photons are lost from each pulse. Eve's attempt to measure the leaked parts of a signal's wave function and obtain information about the sent bit would decrease the entropy. However, extracting a substantial amount of information from leaked photons requires Eve to operate and observe multitudes of degrees of freedom, which, as we will demonstrate in the next section, is unfeasible.

1.1 Required length of the eavesdropping device

In assessing the feasibility of the potential eavesdropping on the natural losses, we consider a scheme in which logical bits are encoded into optical coherent states $|\gamma_0\rangle$ and $|\gamma_1\rangle$ with different photon numbers $\mu_0 = |\gamma_0|^2$ and $\mu_1 = |\gamma_1|^2$. Let the signal pulses have the duration of 1 ns (0.2 m long) and comprise 10^4 photons on average. The optimal values for μ_0 and μ_1 on a 1000 km line, as determined by our simulations (see the main text), are 9000 and 11,000, respectively. To eavesdrop on the homogeneously spread natural losses, an eavesdropper would be forced to undertake measurements along various segments of the fiber, possibly using single-photon detectors. However, as our calculations indicate, such a method would be impractical in terms of the sheer length required to successfully determine the value of any given bit.

The natural losses coefficient of a fiber section of length l can be calculated as

$$r_l = 1 - 10^{-\xi \cdot l}, \tag{1}$$

where ξ is the decay constant. The number of photons lost from a wave packet containing μ_a photons is given by:

$$\mu_{\mathrm{E}}^{(a)} = \mu_a \cdot r_l. \tag{2}$$

The lower index E denotes that the photons can be seized by an eavesdropper, the upper index a represents the corresponding random bit value.

The observable (positive operator-valued measure, or POVM) describing the single-photon detector includes two projective operators corresponding to two possible outcomes:

$$\mathcal{M}_{\text{single photon}} = \left\{ \hat{M}_0 = |0\rangle\langle 0|\,,\ \hat{M}_{\text{click}} = \sum_{n=1}^{+\infty} |n\rangle\langle n| \right\}. \tag{3}$$

The probability of the detector's "click" conditional to the bit value a is determined by the Poisson statistics of Eve's coherent state

$$\begin{aligned} q_a \equiv p\,(\text{click}\,|\,a) &= \mathrm{Tr}\left(\hat{M}_{\text{click}} \cdot |\sqrt{r_l}\gamma_a\rangle\langle\sqrt{r_l}\gamma_a|\right) \\ &= 1 - \mathrm{Tr}\left(\hat{M}_0 \cdot |\sqrt{r_l}\gamma_a\rangle\langle\sqrt{r_l}\gamma_a|\right) = 1 - \left|\langle 0|\sqrt{r_l}\gamma_a\rangle\right|^2 \\ &= 1 - e^{-|\sqrt{r_l}\gamma_a|^2} = 1 - e^{-\mu_{\mathrm{E}}^{(a)}}. \end{aligned} \tag{4}$$

According to the measurement outcomes, Eve makes bit decisions. Probability distribution of measurement results can be considered as Binomial which variance is $q_a(1-q_a)$. Carrying out N independent measurements of sequential parts of the line, the combined variance is a sum of variances of each individual measurement. Thus, the expression for the square root of the variance takes form

$$\delta n_a = \sqrt{N \cdot q_a(1-q_a)}. \tag{5}$$

Bits zero and one produce different distributions, the distance between the maximums of these distributions can be calculated as

$$\Delta n = N \cdot \left|\mu_{\mathrm{E}}^{(1)} - \mu_{\mathrm{E}}^{(0)}\right|. \tag{6}$$

In order to obtain significant amount of information Eve needs the distance between the maximums of the distributions to exceed the sum of their standard derivations, i.e. the notional critical condition can be written as

$$\begin{aligned} &\Delta n = \delta n_0 + \delta n_1 \quad \Rightarrow \\ &\qquad N \cdot \left|\mu_{\mathrm{E}}^{(1)} - \mu_{\mathrm{E}}^{(0)}\right| = \sqrt{N} \cdot \left(\sqrt{q_0(1-q_0)} + \sqrt{q_1(1-q_1)}\right). \end{aligned} \tag{7}$$

Then, supposing that each of the detectors covers a piece of fiber of the length equal to the length of the considered pulses, i.e. $l = 0.2$ m, and taking $\xi = 0.02\,\mathrm{km}^{-1}$, which is a common value for the single-mode optical fiber, the required number of detectors

$$N = \frac{\left(\sqrt{q_0(1-q_0)} + \sqrt{q_1(1-q_1)}\right)^2}{\left|\mu_{\mathrm{E}}^{(1)} - \mu_{\mathrm{E}}^{(0)}\right|^2} \simeq 10^3. \tag{8}$$

Combining all measured pieces, we obtain the total length of the whole detection device $N \cdot l \simeq 10^3 \cdot 0.2\,\mathrm{m} = 200\,\mathrm{m}$.

Moreover, not all the leakages can be measured in reality: some of the scattered photons transform to different modes propagating along the fiber and do not radiate outwards. Along with that, to measure a leaked

part of the signal with the single photon detector, it is necessary to isolate the measured part of the line from the external radiation and concentrate the leaked photons to the cryogenic setup. The effects combined reduce the number of photons available to Eve approximately by an order of magnitude. These physically motivated assumptions lead to enormous lengths of detection devices even in the case of higher intensities (for instance, $\mu_{0,1} \sim 10^5$, which turned out to be optimal for the 40 000 km line – see Numerical Simulations). It is important to acknowledge that while the aforementioned attacks utilizing scattering losses are hardly realistic, one can employ methods of protection that can counteract them as well. These include utilizing specialized cable design enabling the controlled dissipation of scattering losses (described in Note 2), utilizing lower numbers of photons in pulses, and implementing advanced encoding schemes that utilize the phase or shape of the pulses.

1.2 Precise estimation of Eve's information

To estimate the precise amount of information that Eve can get from the N individual measurements of natural losses with single photon detectors, we calculate the mutual information between Alice's sent bit $a \in \{0, 1\}$ and Eve's measurement results. The conditional probabilities of obtaining n "clicks" can be written as

$$p(n|a) = C_N^n \cdot q_a^n \cdot (1 - q_a)^{N-n}, \tag{9}$$

where $C_N^n = \frac{N!}{n!(N-n)!}$ is a binomial coefficient and q_a is the click probability in the individual measurement in the case where the sent bit value is a. The expression for the mutual information is determined by the joint probability distribution $p(n, a) = p(n|a) \cdot p(a)$:

$$\begin{aligned} I\left(\mathrm{A} : \mathrm{E}_{\mathrm{ind}}^{(N)}\right) &= \sum_{n=0}^{N} \sum_{a=0}^{1} p(n, a) \cdot \log_2 \left(\frac{p(n, a)}{p(n) \cdot p(a)} \right) \\ &= \sum_{n=0}^{N} \sum_{a=0}^{1} \frac{1}{2} p(n|a) \cdot \log_2 \left(\frac{p(n|a)}{p(n|0) + p(n|1)} \right) \\ &= 1 - \frac{1}{2} \sum_{n=0}^{N} \left(p(n|0) + p(n|1) \right) \cdot h_2 \left(\frac{p(n|0)}{p(n|0) + p(n|1)} \right). \end{aligned} \tag{10}$$

The dependence of the Eq. (10) on the total length of the detection device is depicted in Fig. 1. Was the device's length equal to 200 m, the mutual information $I\left(\mathrm{A} : \mathrm{E}_{\mathrm{ind}}^{(N)}\right)$ would almost reach 0.5, meaning that Eve would know half of the raw shared key—these results are in a good agreement with the preliminary estimations from the previous section. While in the case of more feasible lengths (a couple of meters, for example), the information Eve can obtain is of the order of 10^{-2} bit. One can note that the Eq. (10) does not depend on whether phase randomization is applied or not, since the probabilities are determined only by photon numbers (for details see Note 4).

1.3 Ideal photon number measurement

Next, we consider collective photon number measurement over the whole N pieces of the fiber which can be conducted after gathering all the scattered photons at one ideal detector. The corresponding observable in terms of the POVM effects can be expressed as projectors on the Fock-states

$$\mathcal{M}_{\text{photon number}} = \left\{ \hat{M}_n = |n\rangle \langle n| \right\}_{n=0}^{+\infty}. \tag{11}$$

The probability of obtaining k photons is determined by the average number of all scattered photons in a signal corresponding to sent bit a

$$p(k|a) = e^{-N \cdot \mu_{\mathrm{E}}^{(a)}} \cdot \frac{\left(N \mu_{\mathrm{E}}^{(a)}\right)^k}{k!} \tag{12}$$

Almost analogously to the previous paragraph, one can calculate the mutual information as

$$I\left(\mathrm{A} : \mathrm{E}_{\mathrm{col}}^{(N)}\right) = 1 - \frac{1}{2} \sum_{k=0}^{+\infty} \left(p(k|0) + p(k|1) \right) \cdot h_2 \left(\frac{p(k|0)}{p(k|0) + p(k|1)} \right). \tag{13}$$

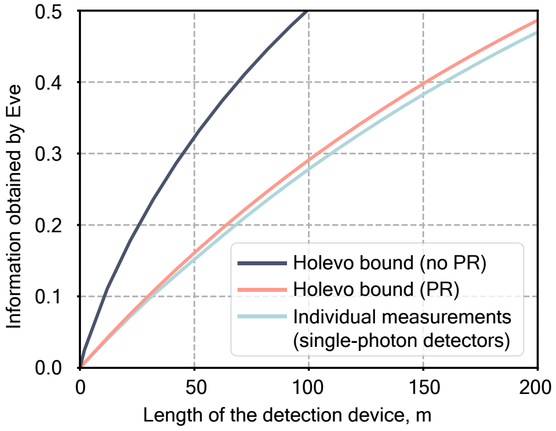

Figure 1 | The information Eve can obtain from natural losses as a function of the overall length of the detection device. The cyan line corresponds to individual measurements (by single-photon detectors) as determined by Eq. (10). The orange line depicts Holevo bound with phase randomization (PR) according to Eq. (18). The black line is for the Holevo bound (no PR) in the absence of PR Eq. (16).

The only difference from the Eq. (10) is that upper limit of the summation is now infinity. As depicted in the Fig. 1, the informational advantage of Eq. (13) over single-photon measurements is insignificant.

1.4 The Holevo bound

To build an upper-bound on the information that Eve may extract from the scattered photons, we calculate the Holevo quantity, which for an ensemble of quantum states $\mathcal{E} = \left\{ \left(1/2, \hat{\rho}^{(0)}\right), \left(1/2, \hat{\rho}^{(1)}\right) \right\}$ is defined as

$$\chi(\mathcal{E}) = S\left(\frac{1}{2}\hat{\rho}^{(0)} + \frac{1}{2}\hat{\rho}^{(1)} \right) - \frac{1}{2} S\left(\hat{\rho}^{(0)}\right) - \frac{1}{2} S\left(\hat{\rho}^{(1)}\right), \tag{14}$$

where $S(\hat{\rho}) = -\mathrm{Tr}\,(\hat{\rho} \cdot \log_2 \hat{\rho})$ is von Neuman entropy. Without phase randomization, Eve has to distinguish between pure coherent states of the form

$$\hat{\rho}^{(a)} = |\sqrt{r_l N \gamma_a}\rangle \langle \sqrt{r_l N \gamma_a}|. \tag{15}$$

Since entropy of a pure state is zero, the Holevo quantity is just the entropy of the average ensemble's state

$$\begin{aligned} \chi(\mathcal{E}) = S\left(\frac{1}{2}\hat{\rho}^{(0)} + \frac{1}{2}\hat{\rho}^{(1)} \right) &= h_2 \left(\frac{1}{2} - \frac{1}{2} \left| \langle \sqrt{r_l N \gamma_0} | \sqrt{r_l N \gamma_1} \rangle \right| \right) \\ &= h_2 \left(\frac{1}{2} - \frac{1}{2} \exp\left[-\frac{1}{2} \cdot r_l N (\gamma_1 - \gamma_0)^2 \right] \right), \end{aligned} \tag{16}$$

where $h_2(x) = x \cdot \log_2(x) - (1-x) \cdot \log_2(1-x)$ is binary entropy. Applying phase randomization transforms the ensemble of pure coherent states into the mixtures of Fock states

$$\begin{aligned} \mathcal{E}_{\mathrm{PR}} &= \left\{ \left(\frac{1}{2}, \hat{\rho}_{\mathrm{PR}}^{(0)} \right), \left(\frac{1}{2}, \hat{\rho}_{\mathrm{PR}}^{(1)} \right) \right\}, \\ \hat{\rho}_{\mathrm{PR}}^{(a)} &= \frac{1}{2\pi} \int_0^{2\pi} d\varphi \, |\sqrt{r_l N \gamma_a} \cdot e^{i\varphi}\rangle \langle \sqrt{r_l N \gamma_a} \cdot e^{i\varphi}| = \sum_{k=0}^{+\infty} p(k|a) \cdot |k\rangle\langle k|, \end{aligned} \tag{17}$$

where $p(k|a)$ is defined in Eq. (12). Now ensemble's states are diagonal, thus, quantum entropy is replaced with classical Shannon entropy

$$\begin{aligned} \chi(\mathcal{E}_{\mathrm{PR}}) &= S\left(\frac{1}{2}\hat{\rho}_{\mathrm{PR}}^{(0)} + \frac{1}{2}\hat{\rho}_{\mathrm{PR}}^{(1)} \right) - \frac{1}{2} S\left(\hat{\rho}_{\mathrm{PR}}^{(0)}\right) - \frac{1}{2} S\left(\hat{\rho}_{\mathrm{PR}}^{(1)}\right) \\ &= H\left(\left\{ \frac{p(k|0) + p(k|1)}{2} \right\}_{k=0}^{+\infty} \right) - \frac{1}{2} \left[H\left(\left\{ p(k|0) \right\}_{k=0}^{+\infty} \right) + H\left(\left\{ p(k|1) \right\}_{k=0}^{+\infty} \right) \right]. \end{aligned} \tag{18}$$

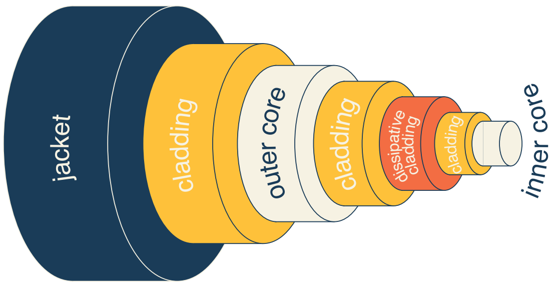

Figure 2 | Cable design. The cable namely includes (i) the inner core for information transmission and physical loss control, (ii) dissipative cladding converting scattering losses from the inner core into heat and completely destructing their information contents, and (iii) outer core for monitoring line integrity.

Carrying out trivial mathematical transformations, one may conclude that the Holevo quantity for the phase randomization case Eq. (18) coincides with the information obtained in ideal photon number measurement Eq. (13). Figure 1 also shows that the Holevo quantity for pure coherent states Eq. (16) appeared to be much higher than considered photon number measurements, meaning that Eve may potentially utilize information about the phase to conduct more effective measurement. It prompts legitimate users to implement phase randomization in their QKD scheme.

NOTE 2. Controlled dissipation of natural losses and advanced line tomography

In this note, we turn our attention to a particular cable design and advanced line tomography that transforms scattered photons into heat in a controlled manner. This method effectively precludes Eve from exploiting the natural losses.

The cable design transforming scattering losses into heat is sketched in Fig. 2. The inner fiber core carrying the information light pulses is surrounded by a cladding with a smaller refractive index and then by the dissipative cladding made out of metal or metal-doped silica. The dissipative cladding screens the scattering losses escaping the fiber core since the scattered wave undergoes the inelastic secondary scattering and transforms into heat. The resulting dissipative thermal losses cannot be deciphered even in principle. The dissipative cladding is, in turn, coated by the second standard refractive cladding layer, surrounded by an outer hollow fiber core and the outside refractive cladding. The outer core enables legitimate users to perform constant reflectometry and transmittometry, thus exercising control over the dissipation of the scattering losses. To remove the dissipative cladding for collecting the scattering losses or to create an artificial leakage from the inner core, Eve first needs to get through the outer core. The action, however, will not go unnoticed because of the permanent Alice and Bob's updating of the outer core's loss profile. Finally, the structure is surrounded by an outer jacket which may include a strengthening layer. To mitigate local losses on fiber connections, they should be made spliced.

Physical loss control allows for a quantitative estimate and localization of any damage inflicted upon the outer or inner cores, see an experimentally obtained example of a reflectogram in Fig. 4 of the main text, presenting the dependence of the backscattered power upon the distance to the scattering point: the linear regions represent homogeneous scattering along the line, while the sharp peaks or drops correspond to the local losses at fiber contacts and bends. As opposed to the regular optical fiber, this specific line design eliminates the possibility of an undetected collection of scattering losses. Any local intrusion brings Eve a negligible number of scattered photons; then, to collect a sufficient amount of the scattering losses, she needs to breach a large section of the controlled outer core, and before it is done, the legitimate users terminate the protocol. Yet, our approach—particularly, the protocol outlined in the main text—resists a significant signal leakage fraction without the necessity of terminating transmission at first sight of the channel breach. Instead, the legitimate users adapt the encoding and post-processing parameters based on the evaluation of the fraction of the signal possibly leaked to Eve, the outer and inner cores being monitored separately. Importantly, although the inner core of the line is controlled only at step 1 of the protocol, legitimate users must control the outer core at all steps of the protocol. The users can count scattering losses that leaked from the breached outer core region as stolen by Eve and then act accordingly, i.e., adapt or terminate the protocol. In other words, the scattering losses escaping the breached outer core region can be equated to the artificial leakages directly from the inner core. If the outer core is breached at the same spot where the inner core has an irredeemable local leakage, such as a bend, the users must take that this leakage is seized by Eve.

NOTE 3. Signal amplification

In this note, we address optical amplification using the formalism of quantum channels. We develop a mathematical representation of a sequence of optical amplifiers, which we later use for modeling a general beam splitter attack on the transmission. Using this representation, we calculate the amplification-induced noise.

3.1 Amplification in doped fibers and losses

In Er/Yt doped fiber, the photonic mode propagates through the inverted atomic medium. To keep the medium inverted, a seed laser of a different frequency co-propagates with the signal photonic mode in the fiber and is then filtered out at the output by means of wavelength-division multiplexing (WDM). The interaction between the inverted atoms and propagating light field mode $\hat{a}$ is given by the Hamiltonian in the rotating wave approximation

$$\hat{H}_{\rm int} = i\kappa \sum_{n=1}^{N} \left(\hat{a}^\dagger \hat{\sigma}_-^{(n)} - \hat{a}\hat{\sigma}_+^{(n)}\right) = i\kappa \left(\hat{a}^\dagger \hat{S}_- - \hat{a}\hat{S}_+\right), \tag{19}$$

$$\hat{\sigma}_-^{(n)} = |0\rangle\langle 1|_n\,, \quad \hat{\sigma}_+^{(n)} = |1\rangle\langle 0|_n\,, \quad \hat{S}_\pm = \sum_{n=1}^{N} \hat{\sigma}_\pm^{(n)}, \tag{20}$$

where we enumerate the atoms by index n, with N being the overall number of atoms in the medium ($N \gg 1$), κ is the interaction constant. Here, each medium's atom is assumed to be a two-level system with its basis states $|0\rangle$ and $|1\rangle$ denoting the ground and excited states, respectively; $\hat{\sigma}_\pm^{(n)}$ is the n-th atom raising/lowering operator, while $\hat{S}_\pm$ is the collective raising/lowering operator, that shifts the number of excited atoms in the medium by one. To simplify further calculations, we utilize the Holstein-Primakoff [82] transformation which provides mapping between the collective $(\hat{S}_+, \hat{S}_-)$ and boson $(\hat{b}, \hat{b}^\dagger)$ operators

$$\hat{S}_+ = \sqrt{N}\sqrt{1 - \frac{\hat{b}^\dagger\hat{b}}{N}}\,\hat{b}, \quad \hat{S}_- = \sqrt{N}\hat{b}^\dagger\sqrt{1 - \frac{\hat{b}^\dagger\hat{b}}{N}}, \tag{21}$$

where $\left[\hat{b}, \hat{b}^\dagger\right] = 1$. In the regime, where average number of excitations is much smaller than the number of atoms $\langle\hat{b}^\dagger\hat{b}\rangle \ll N$, one may use the approximation

$$\hat{S}_+ \approx \sqrt{N}\hat{b}, \quad \hat{S}_- \approx \sqrt{N}\hat{b}^\dagger. \tag{22}$$

The initial state of fully inverted atomic medium is $|1\rangle_1 \otimes |1\rangle_2 \ldots \otimes |1\rangle_N$ (all N atoms are in the excited state). The Holstein-Primakoff transformation maps it to the vacuum $|0\rangle_b$ (i.e. no excitations in the boson mode b). The amplifier's medium with m atoms in the ground state is now described by the state with m excitations $|m\rangle_b$ that obeys the standard annihilation and creation operations

$$\begin{aligned} \hat{b}\,|m\rangle_b &= \sqrt{m}\,|m-1\rangle_b\,, \\ \hat{b}^\dagger\,|m\rangle_b &= \sqrt{m+1}\,|m+1\rangle_b\,. \end{aligned} \tag{23}$$

With that we have

$$\hat{H}_{\mathrm{int}} \approx i\kappa\sqrt{N}\left(\hat{a}^\dagger\hat{b}^\dagger - \hat{a}\hat{b}\right). \quad (24)$$

The evolution operator of a propagating photon is given by

$$\hat{U}_g = e^{-i\hat{H}_{\mathrm{int}}t/\hbar} = e^{g(\hat{a}^\dagger\hat{b}^\dagger - \hat{a}\hat{b})}, \quad (25)$$

where $g = \kappa\sqrt{N}t/\hbar$ and t is effective time of interaction between the photonic mode and atomic medium. Besides considering the channel acting on the propagating state, we also have to consider a conjugate channel acting on the creation operator $\hat{a}$ (it will be needed in the following cryptanalysis)

$$\mathrm{Amp}^*_G[\hat{a}] = \hat{U}^\dagger_g\,\hat{a}\,\hat{U}_g = \cosh(g)\hat{a} + \sinh(g)\hat{b}^\dagger. \quad (26)$$

In practice, the performance of erbium-doped fiber amplifiers (EDFAs) suffers from technical limitations, which arise in addition to the amplification limits on added quantum noise. These limitations are mainly caused by two factors: (i) the atomic population may be not completely inverted throughout the media, (ii) there may be coupling imperfection between the optical mode and the doped fiber section or main part of the fiber. We will imply that these factors are accounted for in the loss channel prior to the amplification channel, as shown in Ref. [83]. The canonical transformation of the loss channel is

$$\mathrm{Loss}^*_T[\hat{a}] = \hat{U}^\dagger_T\hat{a}\hat{U}_T = \sqrt{T}\hat{a} + \sqrt{1-T}\hat{c}, \qquad T = \cos^2\lambda, \quad (27)$$

where λ is the interaction parameter, T is the proportion of the transmitted signal, the annihilation operator $\hat{c}$ corresponds to the initially empty mode which the lost photons go to, and $\hat{U}_\lambda = e^{\lambda\hat{a}^\dagger\hat{c} - \lambda\hat{a}\hat{c}^\dagger}$.

3.2 *P*-function and its evolution under amplification

Consider a single photonic mode with bosonic operators $\hat{a}$ and $\hat{a}^\dagger$ acting in the Fock space. To understand the effect of the amplification on the bosonic mode state, we will use the P-function formalism allowing to express any density operator as a quasi-mixture of coherent states:

$$\hat{\rho} = \int d^2\alpha\,\mathrm{P}(\alpha)\,|\alpha\rangle\langle\alpha|, \quad (28)$$

where $d^2\alpha \equiv d\mathrm{Re}(\alpha)\,d\mathrm{Im}(\alpha)$ and the quasi-probability distribution $\mathrm{P}(\alpha)$ is not necessarily positive. For a given state with the density matrix $\hat{\rho}$ the P-function can be written as

$$\mathrm{P}(\alpha) = \mathrm{tr}:\delta(\hat{a}-\alpha):\hat{\rho}, \quad (29)$$

where

$$:\delta(\hat{a}-\alpha): := \frac{1}{\pi^2}\int d^2\beta\, e^{\alpha\beta^* - \alpha^*\beta} e^{\beta\hat{a}^\dagger} e^{-\beta^*\hat{a}}, \quad (30)$$

see Ref. [84] for details. Amplification is described by a quantum channel given by

$$\mathrm{Amp}_{G=\cosh^2(g)}:\hat{\rho}\mapsto \mathrm{Amp}_G[\hat{\rho}] = \mathrm{tr}_b\left[\hat{U}_g\,\hat{\rho}\otimes|0\rangle\langle 0|_b\,\hat{U}^\dagger_g\right], \quad (31)$$

where $\hat{U}_g$ is defined by Eq. (25), g is the interaction parameter characterizing the amplifier, $G = \cosh^2(g)$ is the factor by which the intensity of the input signal is amplified, and annihilation operator $\hat{b}$ corresponds to the auxiliary mode starting in the vacuum states. see e.g. [85].

To see how the P-function of a state transforms under the optical amplification, consider a simple situation where the input signal is in the pure coherent state $|\gamma\rangle\langle\gamma|$ with the corresponding initial P-function $\mathrm{P}_i(\alpha) = \delta(\alpha-\gamma)$ (delta-function acting on the complex plane). After the amplification the P-function becomes

$$P(\alpha,\gamma,g) = \mathrm{tr}:\delta(\hat{a}-\alpha):\mathrm{Amp}_G\left[|\gamma\rangle\langle\gamma|\right]. \quad (32)$$

Bearing in mind the canonical transformation of the amplifier channel from Eq. (26), we get Eq. (3) from the main text: given a pure coherent input state (with complex amplitude γ), the output state is a mixture of normally distributed coherent states; the mean complex amplitude is $\sqrt{G}\gamma$ and the standard deviation is $(G-1)/\sqrt{2}$:

$$P(\alpha,\gamma,G) = \frac{1}{\pi(G-1)}\exp\left(-\frac{|\alpha-\sqrt{G}\gamma|^2}{G-1}\right). \quad (33)$$

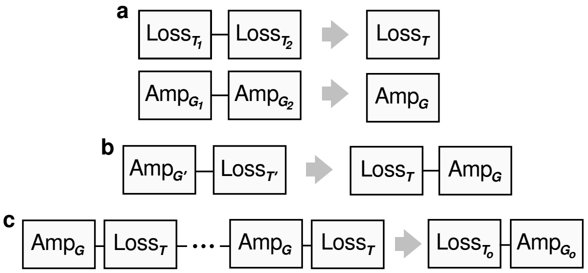

Figure 3 | Compositions of loss and amplification channels and their equivalent representations. a Two loss or amplification channels can be reduced to one. **b** Loss and amplification channels can be effectively rearranged. **c** A series of losses and amplifiers can be reduced to one pair of loss and amplification.

3.3 Composition of amplifiers and losses

In our quantum key distribution (QKD) protocol, the amplification is used to compensate the fiber losses. Long-distance transmission requires a cascade of amplifiers, in which case the signal's evolution is determined by a sequence of multiple loss and amplification channels. In this section we prove that any such sequence can be mathematically reduced to a composition of one loss and one amplification channels.

Statement 1. *Two loss or amplification channels can be effectively reduced to one.*

First, we show that a pair of loss or amplification channels can be effectively reduced to the one channel (Fig. 3**a**). To that end, let us consider two consequent loss channels:

$$\left(\mathrm{Loss}_{T_2}\circ\mathrm{Loss}_{T_1}\right)^*[\hat{a}] = \sqrt{T_1T_2}\hat{a} + \sqrt{T_1(1-T_1)}\hat{c}_1 + \sqrt{1-T_2}\hat{c}_2 = \sqrt{T_1T_2}\hat{a} + \sqrt{1-T_1T_2}\hat{c}, \quad (34)$$

where we defined operator $\hat{c}$

$$\hat{c} = \frac{\sqrt{T_2(1-T_1)}\hat{c}_1 + \sqrt{1-T_2}\hat{c}_2}{\sqrt{1-T_1T_2}}, \quad (35)$$

acting on the vacuum state and satisfying the canonical commutation relation $[\hat{c},\hat{c}^\dagger] = 1$. The last expression of Eq. (34) represents the action of one loss channel with the effective parameter $T = T_1T_2$

$$\mathrm{Loss}_{T_2}\circ\mathrm{Loss}_{T_1} = \mathrm{Loss}_{(T=T_1T_2)}. \quad (36)$$

The same reasoning applies to amplifiers

$$\mathrm{Amp}_{G_2}\circ\mathrm{Amp}_{G_1} = \mathrm{Amp}_{G=G_1G_2}. \quad (37)$$

Statement 2. *Loss and amplification can always be represented as a composition where loss is followed by amplification.*

Let us show that the composition of an amplification channel followed by a loss channel can be mathematically replaced with the pair of certain loss and amplification channels acting in the opposite order (Fig. 3**b**). Firstly, let us consider the transformation corresponding to the amplification followed by the loss

$$\left(\mathrm{Loss}_{T'}\circ\mathrm{Amp}_{G'}\right)^*[\hat{a}] = \hat{U}^\dagger_{g'}\hat{U}^\dagger_{\lambda'}\hat{a}\,\hat{U}_\lambda\hat{U}_g = \sqrt{T'G'}\hat{a} + \sqrt{1-T'}\hat{c} + \sqrt{T'(1-G')}\hat{b}^\dagger. \quad (38)$$

In the case of the opposite order we obtain

$$(\mathrm{Amp}_G \circ \mathrm{Loss}_T)^*[\hat{a}] = \hat{U}_\lambda^\dagger \hat{U}_g^\dagger \hat{a}\, \hat{U}_g \hat{U}_\lambda = \\ = \sqrt{TG}\hat{a} + \sqrt{G(1-T)}\hat{c} + \sqrt{G-1}\hat{b}^\dagger. \quad (39)$$

Considered transformations become identical when the parameters are related as

$$\mathrm{Loss}_{T'} \circ \mathrm{Amp}_{G'} = \mathrm{Amp}_G \circ \mathrm{Loss}_T, \\ T = \frac{G'T'}{(G'-1)T'+1}, \\ G = (G'-1)T'+1. \quad (40)$$

In other words, the two types of channels ”commute” provided that the parameters are modified in accord with these relations. In particular, the parameters in the equation above are always physically meaningful $G \geq 1$, $0 \leq T \leq 1$, meaning that we can always represent loss and amplification in form of a composition where loss is followed by amplification (the converse is not true).

Statement 3. *A series of losses and amplifiers can be effectively reduced to one pair of loss and amplification.*

Let us finally show that the sequence of loss and amplification channels can be mathematically represented as one pair of loss and amplification (Fig.3**c**). Consider the transformation

$$\Phi_M = (\mathrm{Amp}_G \circ \mathrm{Loss}_T)^{\circ M}, \quad (41)$$

corresponding to the series of M identical loss and amplification channels, for which we want to find a simple representation. According to Statement 2, we can effectively move all losses to the right end of the composition, i.e., permute the channels in such a way that all the losses act before amplification. Every time the loss channel with the transmission probability $T_{(i)}$ is moved before an amplifier with the amplification factor $G_{(i)}$, the parameters are transformed in accord with Eq. (40):

$$T_{(i)} \mapsto T_{(i+1)} = \frac{G_{(i)}T_{(i)}}{(G_{(i)}-1)T_{(i)}+1}, \\ G_{(i)} \mapsto G_{(i+1)} = (G_{(i)}-1)T_{(i)}+1. \quad (42)$$

In our sequence we can pairwise transpose all neighboring losses with amplifier (starting with the first amplifier and the second loss). After repeating this operation $M-1$ times, bearing in mind the Statement 1, we find that

$$\Phi_M = \mathrm{Amp}_{G_{(0)}} \circ \mathrm{Amp}_{G_{(1)}} \circ \cdots \circ \mathrm{Amp}_{G_{(M-1)}} \\ \circ \mathrm{Loss}_{T_{(M-1)}} \circ \mathrm{Loss}_{T_{(M-2)}} \circ \cdots \circ \mathrm{Loss}_{T_{(0)}} = \mathrm{Amp}_{G_\circ} \circ \mathrm{Loss}_{T_\circ}, \quad (43)$$

where

$$T_\circ = \prod_{i=0}^{M-1} T_{(i)}, \quad G_\circ = \prod_{i=0}^{M-1} G_{(i)}, \quad (44)$$

i.e., the series of losses and amplifiers is equivalent to the loss channel of transmission $T_\circ$ followed by the amplifier with amplification factor $G_\circ$. Note now that the value $\eta \equiv G_{(i)}T_{(i)} = GT$ cannot be changed by permutations. Let us define

$$F_{(i)} = (G_{(i)}-1)T_{(i)}+1, \quad (45)$$

and bear in mind that

$$F_{(i+1)} = (G_{(i+1)}-1)T_{(i+1)}+1 = \frac{(F_{(i)}-1)}{F_{(i)}}TG+1 = \eta\left(\frac{F_{(i)}-1}{F_{(i)}}\right)+1. \quad (46)$$

We can write

$$T_{(i+1)} = \frac{TG}{F_{(i)}}, \quad G_{(i+1)} = F_{(i)}, \quad (47)$$

and

$$G_\circ = G\prod_{i=0}^{M-2} F_{(i)}, \quad T_\circ = \frac{T(TG)^{M-1}}{\prod_{i=0}^{M-2} F_{(i)}} = \frac{(TG)^M}{G_\circ}. \quad (48)$$

Let us find the explicit form of $G_\circ$ and $T_\circ$ by solving the recurrence relation. Define A_n and B_n through the relation

$$F_{(n-1)} = \frac{A_n}{B_n}. \quad (49)$$

Then

$$F_{(n+1)} = \frac{(\eta+1)F_{(n)}-\eta}{F_{(n)}} = \frac{(\eta+1)A_{n+1}-\eta B_{n+1}}{A_{n+1}}. \quad (50)$$

It follows from Eqs. (49) and (50) that $B_{n+1} = A_n$ and

$$A_{n+1} = (\eta+1)A_n - \eta B_n = (\eta+1)A_n - \eta A_{n-1}. \quad (51)$$

We see that the solution of this equation has a form

$$A_n = c_1 + c_2\eta^n, \quad (52)$$

where c_1 and c_2 are the constants, which are determined by $F_0 = (G-1)T+1$: we take $A_1 = (G-1)T+1$ and $A_0 = 1$, and obtain

$$c_1 = \frac{T-1}{GT-1}, \quad c_2 = \frac{(G-1)T}{GT-1}. \quad (53)$$

Notably, the product $\Pi_{n=0}^{M-2}F_{(n)}$ appearing in the final expression becomes relatively simple

$$\prod_{n=0}^{M-2} F_{(n)} = \frac{(G-1)(GT)^M + G(T-1)}{G(GT-1)}, \quad (54)$$

and we have

$$\Phi_M = (\mathrm{Amp}_G \circ \mathrm{Loss}_T)^{\circ M} = \mathrm{Amp}_{G_\circ} \circ \mathrm{Loss}_{T_\circ}, \\ G_\circ = \frac{(G-1)(GT)^M + G(T-1)}{GT-1}, \\ T_\circ = \frac{(TG)^M}{G_\circ}. \quad (55)$$

The case of $TG = 1$ is particularly interesting as the average photon number of the transmitted signal remains preserved (which is different from the total output photon number as it has the noise contribution). In the limit $G \to 1/T$ we have

$$G_\circ = G(M(1-T)+T), \\ T_\circ = \frac{T}{M(1-T)+T}. \quad (56)$$

3.4 Effective model of the line

We consider how Eve performs the beam splitter attack seizing the part of the signal somewhere along the optical line as shown in Fig.4**a**. If the signal intensity incident to the beam splitter is 1, then intensity r_E goes to Eve, and $1-r_\mathrm{E}$ goes to Bob's direction. The proportion of the transmitted signal on the distance d between two neighbouring amplifiers is determined by

$$T = 10^{-\xi\cdot d}, \quad G = \frac{1}{T}, \quad (57)$$

where $\xi = 0.02\,\mathrm{km}^{-1}$ is the parameter of losses typical for the optical fibers and G is amplification factor of each amplifier. Let $D_{\mathrm{AB(AE)}}$ be the distance between Alice and Bob (Alice and Eve), then the numbers of amplifiers before and after the beam splitter M_1 and M_2 are given by

$$M_1 = D_{\mathrm{AE}}/d, \\ M_2 = (D_{\mathrm{AB}} - D_{\mathrm{AE}})/d. \quad (58)$$

According to Statement 3, the scheme can be simplified by reducing the losses and amplifications before and after the beam splitter to two loss and amplification pairs with the parameters $\{T_1, G_1\}$ and $\{T_2, G_2\}$ respectively (Fig. 4**b**)

$$G_1 = G(M_1(1-T)+T) = \left(10^{-\xi d}-1\right)\cdot\frac{D_{\mathrm{AE}}}{d}+1, \; T_1 = \frac{1}{G_1}, \quad (59)$$

$$G_2 = G(M_2(1-T)+T) = \left(10^{-\xi d}-1\right)\cdot\frac{D_{\mathrm{AB}}-D_{\mathrm{AE}}}{d}+1, \; T_2 = \frac{1}{G_2}. \quad (60)$$

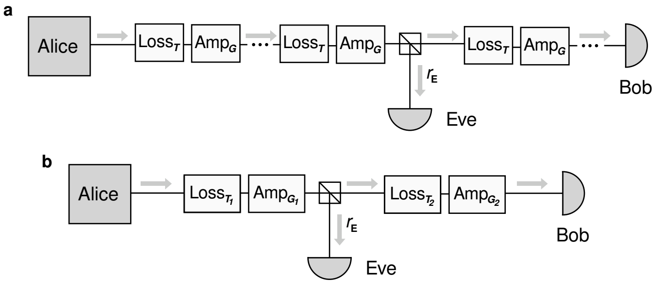

Figure 4 | Schematic representation of a beam splitter attack. a Alice and Bob are connected by a quantum channel comprised of the composition of amplifiers and losses. Eve conducts a beam splitter attack seizing a part of the signal somewhere along the optical line. Thus, she divides the line into two parts. **b** An equivalent scheme: the losses and amplifiers before and after the point of Eve's intervention are represented by two pairs of loss and amplification channels defined by the parameters $\{T_1, G_1\}$ and $\{T_2, G_2\}$ respectively.

3.5 Fluctuations

Let us calculate the fluctuation of the number of photons in a pulse after it passes through a sequence of M loss regions and amplifiers. Let $|\gamma|^2$ be the input average photon number; as follows from Eqs. (28) and (33), the average number of photons n in the output signal is

$$n = \langle \hat a^\dagger \hat a\rangle = |\gamma|^2 + G_\circ - 1, \tag{61}$$

where $G_\circ$ is given by Eq. (56). The variance of the output photon number is

$$\begin{aligned}\delta n &= \sqrt{\langle(\hat a^\dagger \hat a)^2\rangle - (\langle \hat a^\dagger \hat a\rangle)^2}\\ &= \Big(M(G-1)(M(G-1)+1) + |\gamma|^2(2M(G-1)+1)\Big)^{1/2}\end{aligned} \tag{62}$$

Note that even if $|\gamma|^2 = 0$, n and δn are still non-zero. This particularly means that on top of the mode of interest, amplification also generates noise in other modes. Assume that Bob has an optical filter with bandwidth $\Delta\nu$ on which the amplification factor is constant, and the detection time is $\tau \gg 1/\Delta\nu$. Then, the average number of photons due to noise from the secondary modes is

$$n_{\rm noise} \simeq 2[G(M(1-T)+T)-1]\tau\Delta\nu, \tag{63}$$

where factor 2 is due to two possible polarizations. We will thoroughly address the issue of noise at different optical filtration regimes in our forthcoming experimental publication.

In the limit $|\gamma|^2 \gg GM \gg 1$ for the ideal optical filter transmitting only the signal mode we get

$$\delta n \simeq \sqrt{nGM}. \tag{64}$$

Provided that there are no other sources of noise, this quantity determines the precision of the physical loss control: if the test pulse carries n photons, the minimum detectable leakage is

$$r_{\rm E}^{\rm min} \sim \sqrt{MGn}/n = \sqrt{MG/n}. \tag{65}$$

The result coincides with the estimate given in Methods.

NOTE 4. Photon number encoding

This note is devoted to the detailed description of the photon number encoding scheme in the context of the beam splitter attack. We derive the respective amounts of information that the users and Eve know about the transmitted signal. We also address correlations due to the optical amplification between Eve's and Bob's quantum states.

4.1 Phase randomization

In the photon number encoding, we assume the phase of each signal pulse being completely random [86], and unknown both to the eavesdropper and the users. The phase alteration between consecutive signal pulses may be achieved through rebooting the light source after each pulse, or with an additional randomly acting phase modulator. We take that Bob's performs solely the energy measurement of the incoming states without measuring the phases, so Alice does not send the phase reference. In this case, Eve cannot measure the phases either, and the combined system of Alice's random bit (A), the signal incident to Bob (S) and Eve's seized state (E) can be expressed as a mixture of states averaged over all possible phases,

$$\begin{aligned}\left\langle \hat\rho^{\to\rm Bob}_{\rm ASE}\right\rangle_\varphi = \frac{1}{2\pi}\int_0^{2\pi} d\varphi \Bigg[\frac12 \sum_{a=0,1}|a\rangle\langle a|_{\rm A}\otimes \int d^2\alpha\, P(\alpha, \sqrt{T_1}e^{i\varphi}\gamma_a, G_1)\\ \times\left(\int d^2\beta\, P\left(\beta, \sqrt{(1-r_{\rm E})T_2}\alpha, G_2\right)\cdot|\beta\rangle\langle\beta|_{\rm S}\right)\otimes|\sqrt{r_{\rm E}}\alpha\rangle\langle\sqrt{r_{\rm E}}\alpha|_{\rm E}\Bigg].\end{aligned} \tag{66}$$

It follows from Eq. (33) that for any $\varphi \in \mathbb{R}$ we have $P\left(x, ye^{i\varphi}, z\right) = P\left(xe^{-i\varphi}, y, z\right)$. We thus can write

$$\begin{aligned}\left\langle \hat\rho^{\to\rm Bob}_{\rm ASE}\right\rangle_\varphi = \frac12 \sum_{a=0,1}|a\rangle\langle a|_{\rm A}\otimes \int d^2\alpha\, P(\alpha, \sqrt{T_1}\gamma_a, G_1)\\ \times \int d^2\beta\, P\left(\beta, \sqrt{(1-r_{\rm E})T_2}\alpha, G_2\right)\\ \times\frac{1}{2\pi}\int_0^{2\pi} d\varphi\ |e^{i\varphi}\beta\rangle\langle e^{i\varphi}\beta|_{\rm S}\otimes|\sqrt{r_{\rm E}}e^{i\varphi}\alpha\rangle\langle\sqrt{r_{\rm E}}e^{i\varphi}\alpha|_{\rm E}.\end{aligned} \tag{67}$$

4.2 Bob's information

We first estimate the mutual information between Alice and Bob, which is $S(\mathrm{A}) - S(\mathrm{A}|\mathrm{B})$ in Eq. (6) of the main text. For that purpose, we trace out Eve's subsystem and consider a bipartite quantum state shared between legitimate users right before Bob's measurement is conducted $\left\langle\hat\rho^{\to\rm Bob}_{\rm AS}\right\rangle_\varphi = \mathrm{tr}_{\rm E}\left\langle\hat\rho^{\to\rm Bob}_{\rm ASE}\right\rangle_\varphi$

$$\begin{aligned}\left\langle \hat\rho^{\to\rm Bob}_{\rm AS}\right\rangle_\varphi = \frac12 \sum_{a=0,1}|a\rangle\langle a|_{\rm A}\otimes \int d^2\alpha\, P(\alpha, \sqrt{T_1}\gamma_a, G_1)\\ \times\left(\int d^2\beta\, P\left(\beta, \sqrt{(1-r_{\rm E})T_2}\alpha, G_2\right)\cdot\frac{1}{2\pi}\int_0^{2\pi} d\varphi\ |\beta e^{i\varphi}\rangle\langle\beta e^{i\varphi}|_{\rm S}\right).\end{aligned} \tag{68}$$

Given that Alice sent bit $a \in \{0, 1\}$, the probability that Bob's measurement outcome is $b \in \{0, 1, \text{fail}\}$ can be written as

$$\begin{aligned}p(b|a) = \int \frac{d^2\alpha}{\pi(G_1-1)}\cdot\exp\left(-\frac{\left|\alpha-\sqrt{G_1T_1}\gamma_a\right|^2}{G_1-1}\right)\int\frac{d^2\beta}{\pi(G_2-1)}\\ \times\exp\left(-\frac{\left|\beta-\sqrt{(1-r_{\rm E})G_2T_2}\alpha\right|^2}{G_2-1}\right)\cdot\frac{1}{2\pi}\int_0^{2\pi}d\varphi\,\langle\beta e^{i\varphi}|\,\hat E_b\,|\beta e^{i\varphi}\rangle.\end{aligned} \tag{69}$$

The probability of finding k photons in the coherent state $|\beta e^{i\varphi}\rangle$ is defined by the Poisson distribution and depends only on $|\beta|$

$$\left|\langle k|\beta e^{i\varphi}\rangle\right|^2 = \frac{|\beta|^{2k}e^{-|\beta|^2}}{k!} = |\langle k|\beta\rangle|^2, \tag{70}$$

which particularly means that for any b $\langle\beta e^{i\varphi}|\,\hat{E}_b\,|\beta e^{i\varphi}\rangle = \langle\beta|\,\hat{E}_b\,|\beta\rangle$. Thus, the probability of results "0" and "1" can be expressed analytically

$$\langle\beta e^{i\varphi}|\,\hat{E}_1\,|\beta e^{i\varphi}\rangle = \langle\beta|\,\hat{E}_1\,|\beta\rangle = \sum_{k=\mu+\theta_2}^{\mu+\theta_4} \frac{|\beta|^{2k} e^{-|\beta|^2}}{k!} = \frac{\Gamma\left(\mu+\theta_4+1, |\beta|^2\right)}{\Gamma(\mu+\theta_4+1)} - \frac{\Gamma\left(\mu+\theta_2, |\beta|^2\right)}{\Gamma(\mu+\theta_2)}, \quad (71)$$

$$\langle\beta|\,\hat{E}_0\,|\beta\rangle = \frac{\Gamma\left(\mu-\theta_1+1, |\beta|^2\right)}{\Gamma(\mu-\theta_1+1)} - \frac{\Gamma\left(\mu-\theta_3, |\beta|^2\right)}{\Gamma(\mu-\theta_3)}, \quad (72)$$

where $\Gamma(z)$ is the Euler gamma function and $\Gamma(z_1, z_2)$ is the incomplete gamma function. Turning again to Eq. (69), we can change the integration order using Fubini's theorem. The analytical integration over $d^2\alpha$ gives us

$$p(b|a) = \frac{e^{-\frac{\gamma_a^2(1-r_{\mathrm{E}})}{G_1+G_2-2-r_{\mathrm{E}}(G_1-1)}}}{\pi\left(G_1+G_2-2-r_{\mathrm{E}}(G_1-1)\right)} \cdot \int_0^{+\infty} d|\beta|\;|\beta| \cdot e^{-\frac{|\beta|^2}{G_1+G_2-2-r_{\mathrm{E}}(G_1-1)}} \times \langle\beta|\,\hat{E}_b\,|\beta\rangle \int_0^{2\pi} d\varphi_\beta\;\exp\left(\frac{2\sqrt{1-r_{\mathrm{E}}}\gamma_a|\beta|\cos\varphi_\beta}{G_1+G_2-2-r_{\mathrm{E}}(G_1-1)}\right), \quad (73)$$

where $\varphi_\beta = \arg(\beta)$. The rightmost integral can be reduced to the modified Bessel function of the first kind:

$$\int_0^{2\pi} d\varphi_\beta\;\exp\left(\frac{2\sqrt{1-r_{\mathrm{E}}}\gamma_a|\beta|\cos\varphi_\beta}{G_1+G_2-2-r_{\mathrm{E}}(G_1-1)}\right) = 2\pi\cdot I_0\left(\frac{2\sqrt{1-r_{\mathrm{E}}}\gamma_a|\beta|}{G_1+G_2-2-r_{\mathrm{E}}(G_1-1)}\right). \quad (74)$$

Thus, we have

$$p(b|a) = \frac{2e^{-\frac{\gamma_a^2(1-r_{\mathrm{E}})}{G_1+G_2-2-r_{\mathrm{E}}(G_1-1)}}}{G_1+G_2-2-r_{\mathrm{E}}(G_1-1)} \int_0^{+\infty} d|\beta|\;|\beta|\cdot\langle\beta|\,\hat{E}_b\,|\beta\rangle \times I_0\left(\frac{2\sqrt{1-r_{\mathrm{E}}}\gamma_a|\beta|}{G_1+G_2-2-r_{\mathrm{E}}(G_1-1)}\right)\cdot e^{-\frac{|\beta|^2}{G_1+G_2-2-r_{\mathrm{E}}(G_1-1)}}. \quad (75)$$

The mutual information between Alice and Bob after the post-selection can be calculated as

$$I(\mathrm{A},\mathrm{B}) \equiv S(\mathrm{A}) - S(\mathrm{A}|\mathrm{B}) = S(\mathrm{A}) + S(\mathrm{B}) - S(\mathrm{AB}) = h_2\left(\sum_{b=0,1}\frac{p(b|0)}{2p_\checkmark}\right) + h_2\left(\sum_{a=0,1}\frac{p(0|a)}{2p_\checkmark}\right) + \sum_{a=0,1}\sum_{b=0,1}\frac{p(b|a)}{2p_\checkmark}\log_2\left(\frac{p(b|a)}{2p_\checkmark}\right), \quad (76)$$

where $h_2(p) = -p\cdot\log_2 p - (1-p)\cdot\log_2(1-p)$ is the binary entropy and the probability of the successful measurement outcome is

$$p_\checkmark = \frac{1}{2}\sum_{a=0,1}\sum_{b=0,1} p(b|a). \quad (77)$$

4.3 Eve's information

To estimate Eve's information (I(A:E) in the Eq. (6) of the main text), we find the explicit form of the quantum state owned by Eve after the users perform post-selection. The density matrix of the joint ABE system is

$$\left\langle\hat{\rho}^f_{\mathrm{ABE}}\right\rangle_\varphi = \sum_{b=0,1}\sum_{a=0,1}\frac{1}{2p(\checkmark|a)}\int d^2\alpha\;P(\alpha,\sqrt{T_1}\gamma_a, G_1) \times |a\rangle\langle a|_{\mathrm{A}} \otimes |b\rangle\langle b|_{\mathrm{B}} \otimes \frac{1}{2\pi}\int_0^{2\pi} d\varphi\;\left|e^{i\varphi}\sqrt{r_{\mathrm{E}}}|\alpha|\right\rangle\left\langle e^{i\varphi}\sqrt{r_{\mathrm{E}}}|\alpha|\right|_{\mathrm{E}} \times \int d^2\beta\;P\left(\beta, \sqrt{(1-r_{\mathrm{E}})T_2}\alpha, G_2\right)\langle\beta|\hat{E}_b|\beta\rangle. \quad (78)$$

For the further calculations it is useful to introduce "conditional" Eve's density matrix, i.e., Eve's density matrix in the case that Alice sent bit a and Bob got a successful measurement outcome:

$$\hat{\rho}^{(a)}_{\mathrm{E}} \equiv \mathrm{tr}_{\mathrm{AB}}\left[\left(2\,|a\rangle\langle a|_{\mathrm{A}}\otimes\hat{\mathbb{1}}\otimes\hat{\mathbb{1}}\right)\cdot\left\langle\hat{\rho}^f_{\mathrm{ABE}}\right\rangle_\varphi\right]. \quad (79)$$

The explicit form of this matrix is

$$\hat{\rho}^{(a)}_{\mathrm{E}} = \frac{1}{p(\checkmark|a)}\int d^2\alpha\;P\left(\alpha;\sqrt{T_1}\gamma_a, G_1\right) \times \int d^2\beta\;P\left(\beta;\sqrt{(1-r_{\mathrm{E}})T_2}\alpha, G_2\right)\langle\beta|\hat{E}_\checkmark|\beta\rangle \times\left[\frac{1}{2\pi}\int_0^{2\pi} d\varphi\;\left|e^{i\varphi}\sqrt{r_{\mathrm{E}}}|\alpha|\right\rangle\left\langle e^{i\varphi}\sqrt{r_{\mathrm{E}}}|\alpha|\right|_{\mathrm{E}}\right], \quad (80)$$

where $\hat{E}_\checkmark \equiv \hat{E}_0 + \hat{E}_1$. To simplify the expression, one can convert integration into Polar coordinates ($\alpha = |\alpha|e^{i\varphi_\alpha}$, $d^2\alpha = |\alpha|\,d|\alpha|\,d\varphi_\alpha$), apply Fubini's theorem, and carry out integration over φ_α analytically

$$\int_0^{2\pi} d\varphi_\alpha\;P\left(|\alpha|e^{i\varphi_\alpha};\sqrt{T_1}\gamma_a, G_1\right) = \frac{2\exp\left(-\frac{|\alpha|^2+|\gamma_a|^2}{G_1-1}\right)}{G_1-1}\cdot I_0\left(\frac{2|\alpha|\gamma_a}{G_1-1}\right), \quad (81)$$

where $I_0(z)$ is the modified Bessel function of the first kind. The same procedure can be carried out for integration over β: bearing in mind that $\langle\beta|\hat{E}_\checkmark|\beta\rangle = \langle\beta|\hat{E}_1|\beta\rangle + \langle\beta|\hat{E}_0|\beta\rangle \equiv f(|\beta|)$ does not depend on φ_β, we have

$$\int_0^{2\pi} d\varphi_\beta\;P\left(|\beta|e^{i\varphi_\beta};\sqrt{(1-r_{\mathrm{E}})T_2}\alpha, G_2\right) = \frac{2\exp\left(-\frac{|\beta|^2+(1-r_{\mathrm{E}})|\alpha|^2}{G_2-1}\right)}{G_2-1}\cdot I_0\left(\frac{2|\beta|\sqrt{1-r_{\mathrm{E}}}|\alpha|}{G_2-1}\right). \quad (82)$$

The rightmost integral in Eq. (80) also can be calculated in the analytical way and expressed in terms of the Fock states $\{|n\rangle\}_{n=0}^{+\infty}$:

$$\frac{1}{2\pi}\int_0^{2\pi} d\varphi\;\left|e^{i\varphi}\sqrt{r_{\mathrm{E}}}|\alpha|\right\rangle\left\langle e^{i\varphi}\sqrt{r_{\mathrm{E}}}|\alpha|\right| = e^{-r_{\mathrm{E}}|\alpha|^2}\sum_{n=0}^{+\infty}\frac{\left(r_{\mathrm{E}}|\alpha|^2\right)^n}{n!}|n\rangle\langle n|. \quad (83)$$

Substituting the results from Eqs. (81–83) into Eq. (80), we get

$$\hat{\rho}^{(a)}_{\mathrm{E}} = \frac{4/p(\checkmark|a)}{(G_1-1)(G_2-1)}\int_0^{+\infty} d|\alpha|\;|\alpha|\exp\left(-\frac{|\alpha|^2+|\gamma_a|^2}{G_1-1}\right)I_0\left(\frac{2|\alpha|\gamma_a}{G_1-1}\right) \times\int_0^{+\infty} d|\beta|\;|\beta|\exp\left(-\frac{|\beta|^2+(1-r_{\mathrm{E}})|\alpha|^2}{G_2-1}\right)I_0\left(\frac{2|\beta|\sqrt{1-r_{\mathrm{E}}}|\alpha|}{G_2-1}\right)\cdot\langle\beta|\hat{E}_\checkmark|\beta\rangle \times\sum_{n=0}^{+\infty}\exp\left(-r_{\mathrm{E}}|\alpha|^2\right)\frac{\left(r_{\mathrm{E}}|\alpha|^2\right)^n}{n!}|n\rangle\langle n|. \quad (84)$$

Note that the resulting density matrix is diagonal in the Fock basis—which is natural given the phase randomization. The diagonal elements of the matrix can be expressed as

$$\langle n|\hat{\rho}_{\mathrm{E}}^{(a)}|n\rangle = \frac{4r_{\mathrm{E}}^{n}\exp\left(-\frac{\gamma_a^2}{G_1-1}\right)}{n!(G_1-1)(G_2-1)p(\checkmark|a)}\int_0^{+\infty} d|\beta|\ |\beta|\cdot f(|\beta|)\exp\left(-\frac{|\beta|^2}{G_2-1}\right)$$
$$\times\int_0^{+\infty} d|\alpha|\ |\alpha|^{2n+1}\exp\left(-|\alpha|^2\cdot\left[\frac{1}{G_1-1}+\frac{1-r_{\mathrm{E}}}{G_2-1}+r_{\mathrm{E}}\right]\right)$$
$$\times I_0\left(\frac{2|\alpha|\gamma_a}{G_1-1}\right)\cdot I_0\left(\frac{2|\beta|\sqrt{1-r_{\mathrm{E}}}|\alpha|}{G_2-1}\right). \quad (85)$$

In order to take integral over $|\alpha|$ analytically, we utilize the fact that the main contribution to the integral comes from $|\alpha|\gg 1$ which enables us to use the asymptotic expansion [87]:

$$I_0(z) = \frac{e^z}{\sqrt{2\pi z}}\left(1+\frac{1}{8z}+O\left(\frac{1}{|z|^2}\right)\right),\ z\in\mathbb{R}. \quad (86)$$

Thus, we have

$$I_0\left(\frac{2|\alpha|\gamma_a}{G_1-1}\right)\cdot I_0\left(\frac{2|\beta|\sqrt{1-r_{\mathrm{E}}}|\alpha|}{G_2-1}\right)$$
$$= \frac{1}{4\pi|\alpha|}\sqrt{\frac{(G_1-1)(G_2-1)}{\gamma_a|\beta|\sqrt{1-r_{\mathrm{E}}}}}\exp\left(2|\alpha|\cdot\left(\frac{\gamma_a}{G_1-1}+\frac{|\beta|\sqrt{1-r_{\mathrm{E}}}}{G_2-1}\right)\right)$$
$$\times\left(1+\frac{1}{16|\alpha|}\left[\frac{G_1-1}{\gamma_a}+\frac{G_2-1}{|\beta|\sqrt{1-r_{\mathrm{E}}}}\right]+O\left(\frac{1}{|\alpha|^2}\right)\right). \quad (87)$$

Utilizing this form, we get

$$\langle n|\hat{\rho}_{\mathrm{E}}^{(a)}|n\rangle \approx \frac{r_{\mathrm{E}}^{n}\exp\left(-\frac{\gamma_a^2}{G_1-1}\right)/p(\checkmark|a)}{\pi\sqrt{\gamma_a|\beta|\sqrt{1-r_{\mathrm{E}}}(G_1-1)(G_2-1)}}\int_0^{+\infty} d|\beta|\ |\beta|\cdot f(|\beta|)$$
$$\times\left(\varkappa_n(|\beta|)+\frac{1}{16}\left[\frac{G_1-1}{\gamma_a}+\frac{G_2-1}{|\beta|\sqrt{1-r_{\mathrm{E}}}}\right]\tilde{\varkappa}_n(|\beta|)\right)\cdot e^{-\frac{|\beta|^2}{G_2-1}}, \quad (88)$$

where we introduced two subsidiary functions $\varkappa_n(|\beta|)$ and $\tilde{\varkappa}_n(|\beta|)$, $n\in\mathbb{N}$: we define

$$\varkappa_n(|\beta|) = \int_0^{+\infty} d|\alpha|\ \frac{|\alpha|^{2n}}{n!}\cdot e^{-A|\alpha|^2+B|\alpha|}$$
$$= \frac{1}{A^{n+1/2}}\cdot\left[\sqrt{\frac{B^2}{4A}}\cdot {}_1F_1\left(n+1,\frac{3}{2},\frac{B^2}{4A}\right)\right.$$
$$\left.+\frac{\Gamma\left(n+\frac{1}{2}\right)}{2\cdot n!}\cdot {}_1F_1\left(n+\frac{1}{2},\frac{1}{2},\frac{B^2}{4A}\right)\right], \quad (89)$$

where ${}_1F_1(x,y,z)$ is the Kummer confluent hypergeometric function (for large values of the third argument we utilize the approximation from Ref. [87]), and

$$A = \frac{1}{G_1-1}+\frac{1-r_{\mathrm{E}}}{G_2-1}+r_{\mathrm{E}}, \quad (90)$$

$$B\equiv B(|\beta|) = \frac{2\gamma_a}{G_1-1}+\frac{2|\beta|\sqrt{1-r_{\mathrm{E}}}}{G_2-1}. \quad (91)$$

Function $\tilde{\varkappa}_n(|\beta|)$ is defined similarly:

$$\tilde{\varkappa}_n(|\beta|) = \int_0^{+\infty} d|\alpha|\ \frac{|\alpha|^{2n-1}}{n!}\cdot e^{-A|\alpha|^2+B|\alpha|}$$
$$= \frac{1}{A^{n-1/2}}\cdot\left[\sqrt{\frac{B^2}{4A}}\cdot\frac{\Gamma\left(n+\frac{1}{2}\right)}{n!}\cdot {}_1F_1\left(n+\frac{1}{2},\frac{3}{2},\frac{B^2}{4A}\right)\right.$$
$$\left.+\frac{1}{2n}\cdot {}_1F_1\left(n,\frac{1}{2},\frac{B^2}{4A}\right)\right]. \quad (92)$$

For $n=0$ the integral from Eq. (92) does not converge—let us address this case individually. Instead considering two terms of the series in Eq. (86), we take into account only the first one, which makes the primary contribution to the sum for large $|\alpha|$; thus, we get

$$\varkappa_0(|\beta|) = \int_0^{+\infty} d|\alpha|\ e^{-A|\alpha|+B|\alpha|} = \frac{\sqrt{\pi}e^{B^2/4A}}{2\sqrt{A}}\mathrm{erfc}\left(\frac{B}{2\sqrt{A}}\right),\quad \tilde{\varkappa}_0(|\beta|)=0. \quad (93)$$

With the beam splitter attack, Eve gets one of two non-equiprobable quantum states: $\hat{\rho}_{\mathrm{E}}^{(0)}$ with probability $q_0=\frac{p(\checkmark|0)}{2p_\checkmark}$ and $\hat{\rho}_{\mathrm{E}}^{(1)}$ with $q_1=\frac{p(\checkmark|1)}{2p_\checkmark}$. The explicit forms of diagonal $\hat{\rho}_{\mathrm{E}}^{(0)}$ and $\hat{\rho}_{\mathrm{E}}^{(1)}$ can be obtained by substituting the results from Eqs. (89–93) into Eq. (88). Eve's ensemble $\mathcal{E}$ can be shortly defined as

$$\mathcal{E} = \left\{\left(q_0,\hat{\rho}_{\mathrm{E}}^{(0)}\right),\ \left(q_1,\hat{\rho}_{\mathrm{E}}^{(1)}\right)\right\}. \quad (94)$$

The maximum information that Eve can obtain about Alice's bit on average is bounded by the Holevo quantity $\chi(\mathcal{E})$:

$$I(\mathrm{A},\mathrm{E}) \le \chi(\mathcal{E}) = S\left(q_0\hat{\rho}_{\mathrm{E}}^{(0)}+q_1\hat{\rho}_{\mathrm{E}}^{(1)}\right)-q_0 S\left(\hat{\rho}_{\mathrm{E}}^{(0)}\right)-q_1 S\left(\hat{\rho}_{\mathrm{E}}^{(1)}\right). \quad (95)$$

Since $\hat{\rho}_{\mathrm{E}}^{(0)}$ and $\hat{\rho}_{\mathrm{E}}^{(1)}$ are diagonal, Eq. (95) can be simplified by replacing von Neuman entropy S with the classical Shannon entropy H

$$S\left(q_0\hat{\rho}_{\mathrm{E}}^{(0)}+q_1\hat{\rho}_{\mathrm{E}}^{(1)}\right) = H\left(\left\{q_0\langle n|\hat{\rho}_{\mathrm{E}}^{(0)}|n\rangle+q_1\langle n|\hat{\rho}_{\mathrm{E}}^{(1)}|n\rangle\right\}_{n=0}^{+\infty}\right), \quad (96)$$

$$S\left(\hat{\rho}_{\mathrm{E}}^{(a)}\right) = H\left(\left\{\langle n|\hat{\rho}_{\mathrm{E}}^{(a)}|n\rangle\right\}_{n=0}^{+\infty}\right), \quad (97)$$

where the Shannon entropy of a probability distribution $\left\{w_j\right\}_j$ is defined as $H\left(\{w_j\}_j\right) = -\sum_j w_j\log_2\left(w_j\right)$.

4.4 Correlations

Stealing a proportion r_{E} of a coherent signal pulse $|\gamma\rangle$ provides Eve with a state $|\sqrt{r_{\mathrm{E}}}\gamma\rangle$ uncorrelated with $|\gamma\rangle$, as the state of the joint system is described by product $|\sqrt{1-r_{\mathrm{E}}}\gamma\rangle\otimes|\sqrt{r_{\mathrm{E}}}\gamma\rangle$. This is, however, not the case when the signal pulse is subject to optical amplification, turning a pure coherent state into a mixture of coherent states: now, by splitting off same r_{E}-fraction of this mixture, Eve gets a correlated state containing more information about the sent bit. Therefore, contrary to one's executions, for Eve standing right next to Alice may be less effective than somewhere further along the line—provided that the noise from the optical amplifiers does not overweight the benefits of correlations. Assuming that Eve performs the beam splitter attack, we quantify the correlation between Eve's and Bob's measurement results by calculating the Pearson correlation coefficient [88,89]

$$R_{\mathrm{BE}} = \frac{\langle\langle n_{\mathrm{B}}n_{\mathrm{E}}\rangle\rangle}{\sigma_{n_{\mathrm{B}}}\cdot\sigma_{n_{\mathrm{E}}}}, \quad (98)$$

where $\langle\langle\ldots\rangle\rangle$ stands for irreducible correlator, $\sigma_{n_{\mathrm{B(E)}}}$ is the standard variance. The values are defined as follows

$$\langle\langle n_{\mathrm{B}}n_{\mathrm{E}}\rangle\rangle = \langle n_{\mathrm{B}}n_{\mathrm{E}}\rangle_{\hat{\rho}_{\mathrm{BE}}}-\langle n_{\mathrm{B}}\rangle_{\hat{\rho}_{\mathrm{B}}}\cdot\langle n_{\mathrm{E}}\rangle_{\hat{\rho}_{\mathrm{E}}}, \quad (99)$$

$$\sigma_{n_{\mathrm{B}}} = \sqrt{\left\langle n_{\mathrm{B}}^2\right\rangle_{\hat{\rho}_{\mathrm{B}}}-\langle n_{\mathrm{B}}\rangle_{\hat{\rho}_{\mathrm{B}}}^2},\quad \sigma_{n_{\mathrm{E}}} = \sqrt{\left\langle n_{\mathrm{E}}^2\right\rangle_{\hat{\rho}_{\mathrm{E}}}-\langle n_{\mathrm{E}}\rangle_{\hat{\rho}_{\mathrm{E}}}^2}. \quad (100)$$

Averaging over a density matrix $\hat{\rho}$ is denoted here as $\langle\ldots\rangle_{\hat{\rho}}$. As follows from Eq. (67), the Bob-Eve density matrix can be written as

$$\hat{\rho}_{\mathrm{BE}} = \int d^2\alpha\ P\left(\alpha,\sqrt{T_1}\gamma,G_1\right)\int d^2\beta\ P\left(\beta,\sqrt{T_2(1-r_{\mathrm{E}})}\alpha,G_2\right)$$
$$\times\int_0^{2\pi}\frac{d\varphi}{2\pi}\ |\sqrt{1-r_{\mathrm{E}}}\alpha e^{i\varphi}\rangle\langle\sqrt{1-r_{\mathrm{E}}}\alpha e^{i\varphi}|_{\mathrm{B}}\otimes|\sqrt{r_{\mathrm{E}}}\alpha e^{i\varphi}\rangle\langle\sqrt{r_{\mathrm{E}}}\alpha e^{i\varphi}|_{\mathrm{E}}, \quad (101)$$

$$\hat{\rho}_{\rm B} = {\rm tr}_{\rm E}\left[\hat{\rho}_{\rm BE}\right], \quad \hat{\rho}_{\rm E} = {\rm tr}_{\rm B}\left[\hat{\rho}_{\rm BE}\right], \tag{102}$$

where the effective amplification coefficients G_1, G_2 and transmission probabilities T_1, T_2 are defined by Eqs. (59) and (60). The average photon numbers of Bob's and Eve's subsystems are

$$\langle n_{\rm B}\rangle_{\hat{\rho}_{\rm B}} = \int d^2\alpha\ P\left(\alpha,\ \sqrt{T_1}\gamma, G_1\right)\int d^2\beta\ P\left(\beta,\ \sqrt{T_2(1-r_{\rm E})}\alpha, G_2\right)\cdot|\beta|^2 = (1-r_{\rm E})\cdot\left(|\gamma|^2+G_1-1\right)+G_2-1, \tag{103}$$

$$\langle n_{\rm E}\rangle_{\hat{\rho}_{\rm E}} = \int d^2\alpha\ P\left(\alpha,\ \sqrt{T_1}\gamma, G_1\right)\cdot|\sqrt{r_{\rm E}}\alpha|^2 = r_{\rm E}\cdot\left(|\gamma|^2+G_1-1\right). \tag{104}$$

The average product value is

$$\langle n_{\rm B}n_{\rm E}\rangle_{\hat{\rho}_{\rm BE}} = r_{\rm E}(G_2-1)\cdot\left(|\gamma|^2+G_1-1\right) + r_{\rm E}(1-r_{\rm E})\cdot\left(2(G_1-1)^2+4|\gamma|^2(G_1-1)+|\gamma|^4\right). \tag{105}$$

Hence, the expression for the irreducible correlator depends only on $|\gamma|^2$, $r_{\rm E}$ and G_1:

$$\langle\langle n_{\rm B}n_{\rm E}\rangle\rangle = r_{\rm E}(1-r_{\rm E})\cdot(G_1-1)\cdot\left(2|\gamma|^2+G_1-1\right). \tag{106}$$

Here, we utilized the fact that $\langle n\rangle_{|\alpha\rangle\langle\alpha|} = |\alpha|^2$. In turn, the variances are obtained using the fact that $\langle n^2\rangle_{|\alpha\rangle\langle\alpha|} = |\alpha|^4+|\alpha|^2$:

$$\sigma_{\rm B}^2 = (1-r_{\rm E})\cdot\left(|\gamma|^2+G_1-1\right)\cdot(1+2(G_2-1)) + (1-r_{\rm E})^2\cdot(G_1-1)\cdot\left(2|\gamma|^2+G_1-1\right)+G_2(G_2-1), \tag{107}$$

$$\sigma_{\rm E}^2 = r_{\rm E}\left(|\gamma|^2+G_1-1\right)+r_{\rm E}^2(G_1-1)\left(2|\gamma|^2+G_1-1\right). \tag{108}$$

Substituting Eqs. (106–108) and Eqs. (59, 60) into Eq. (98) yields the dependence of $R_{\rm BE}$ on $D_{\rm AE}$. Function $R_{\rm BE}\left(D_{\rm AE}\right)$ is monotonically growing which shows that with Eve approaching Bob, their measurement results become more and more correlated. As expected, if $G_1 = 1$—corresponding to the case where Eve is right next to Alice—$R_{\rm BE}\left(D_{\rm AE}\right)$ vanishes, meaning zero correlations.

NOTE 5. Phase encoding

In this note, we study the phase encoding scheme. We perform our analysis along the same lines as in the case of the photon number encoding.

5.1 Bob's information

For self-evident reasons, phase randomization approach is inapplicable in case of phase encoding-based protocol. The density matrix describing the tripartite system right before Bob's measurement is

$$\hat{\rho}_{\rm AS}^{\to\rm Bob} = \frac{1}{2}\sum_{a=0,1}|a\rangle\langle a|_{\rm A}\otimes\int d^2\alpha\ P(\alpha,\ \sqrt{T_1}\gamma_a, G_1) \times\left(\int d^2\beta\ P\left(\beta,\ \sqrt{(1-r_{\rm E})T_2}\alpha, G_2\right)\cdot|\beta\rangle\langle\beta|_{\rm S}\right). \tag{109}$$

Given that Alice sent bit $a\in\{0,1\}$, the probability that Bob's measurement outcome is $b\in\{0,1\}$ can be written as

$$p(b|a) = \int\frac{d^2\alpha}{\pi(G_1-1)}\cdot\exp\left(-\frac{|\alpha-\gamma_a|^2}{G_1-1}\right) \times\int\frac{d^2\beta}{\pi(G_2-1)}\exp\left(-\frac{\left|\beta-\sqrt{1-r_{\rm E}}\alpha\right|^2}{G_2-1}\right)\cdot\langle\beta|\hat{E}_b|\beta\rangle. \tag{110}$$

The overlap between coherent state $|\beta\rangle$ and the state with a particular value of the $\hat{q}-$quadrature is

$$|\langle q|\beta\rangle|^2 = \sqrt{\frac{2}{\pi}}\cdot\exp\left(-2\left({\rm Re}[\beta]-q\right)^2\right). \tag{111}$$

For successful measurement results on the Bob's side we have

$$\langle\beta|\hat{E}_0|\beta\rangle = \frac{1}{2}\int_{\theta_1'}^{\theta_2'}dq\ e^{-2({\rm Re}[\beta]-q)^2}, \quad \langle\beta|\hat{E}_1|\beta\rangle = \frac{1}{2}\int_{-\theta_2'}^{-\theta_1'}dq\ e^{-2({\rm Re}[\beta]-q)^2}. \tag{112}$$

The expression for conditional probabilities $p(b|a)$ can be determined analytically

$$p(b|a) = \frac{1}{2}{\rm erf}\left(\frac{\sqrt{2}\left(\theta_2'+(-1)^{a+b}\cdot\gamma\sqrt{1-r_{\rm E}}\right)}{\sqrt{1+2(G_2-1)+2(1-r_{\rm E})(G_1-1)}}\right) - \frac{1}{2}{\rm erf}\left(\frac{\sqrt{2}\left(\theta_1'+(-1)^{a+b}\cdot\gamma\sqrt{1-r_{\rm E}}\right)}{\sqrt{1+2(G_2-1)+2(1-r_{\rm E})(G_1-1)}}\right). \tag{113}$$

Since $p(\checkmark|0) = p(\checkmark|1)$, the mutual information between Alice and Bob is

$$I({\rm A},{\rm B}) = S({\rm A})-S({\rm A}|{\rm B}) = 1-h_2\left(\frac{p(1|0)}{p_\checkmark}\right). \tag{114}$$

As a result, we obtain the explicit form of the expression $S({\rm A})-S({\rm A}|{\rm B})$ which we substitute into Eg. (6) of the main text.

5.2 Eve's information

The density matrix of the Alice-Bob-Eve system given that Bob obtained conclusive measurement result is

$$\hat{\rho}_{\rm ABE}^{\rm f} = \frac{1}{2}\sum_{a=0,1}\frac{1}{p(\checkmark|a)}|a\rangle\langle a|_{\rm A}\otimes\sum_{b=0,1}|b\rangle\langle b|_{\rm B}\otimes\int d^2\alpha\ P(\alpha,\ \sqrt{T_1}\gamma_a, G_1) \times\int d^2\beta\ P\left(\beta,\ \sqrt{(1-r_{\rm E})T_2}\alpha, G_2\right)\langle\beta|\hat{E}_b|\beta\rangle\,|\sqrt{r_{\rm E}}\alpha\rangle\langle\sqrt{r_{\rm E}}\alpha|_{\rm E}. \tag{115}$$

To estimate the conditional entropy $S({\rm A}|{\rm E})$ one has to calculate the reduced Alice-Eve density matrix by tracing out Bob's subsystem:

$$\hat{\rho}_{\rm AE}^{\rm f} = {\rm tr}_{\rm B}\left[\hat{\rho}_{\rm ABE}^{\rm f}\right] = \frac{1}{2}\sum_{a=0,1}\frac{1}{p(\checkmark|a)}|a\rangle\langle a|_{\rm A}\otimes\int d^2\alpha\ P\left(\alpha,\ \sqrt{T_1}\gamma_a, G_1\right) \times\int d^2\beta\ P\left(\beta,\ \sqrt{(1-r_{\rm E})T_2}\alpha, G_2\right)\langle\beta|\hat{E}_\checkmark|\beta\rangle\,|\sqrt{r_{\rm E}}\alpha\rangle\langle\sqrt{r_{\rm E}}\alpha|_{\rm E}. \tag{116}$$

It can be rewritten as

$$\hat{\rho}_{\rm AE}^{\rm f} = \int d^2\alpha\ Q_\checkmark[\alpha]\cdot\hat{\rho}_{\rm AE}^{\rm f}[\alpha], \tag{117}$$

where

$$Q_\checkmark[\alpha] = \frac{P\left(\alpha;\sqrt{T_1}\gamma, G_1\right)}{p_\checkmark}\int d^2\beta\ P\left(\beta;\alpha\sqrt{(1-r_{\rm E})T_2}, G_2\right)\langle\beta|\hat{E}_\checkmark|\beta\rangle, \tag{118}$$

$$\hat{\rho}_{\rm AE}^{\rm f}[\alpha] = \frac{1}{2}\sum_{a=0,1}|a\rangle\langle a|_{\rm A}\otimes\left|(-1)^a\sqrt{r_{\rm E}}\alpha\right\rangle\left\langle(-1)^a\sqrt{r_{\rm E}}\alpha\right|_{\rm E}. \tag{119}$$

Mutual information between the eavesdropper and Alice after the post-selection procedure is

$$I({\rm A},{\rm E}) = S({\rm A})-S({\rm A}|{\rm E}) = 1-S({\rm A}|{\rm E}). \tag{120}$$

The lower bound on the entropy $S(\mathrm{A|E})$ may be found by exploiting the concavity of conditional quantum entropy

$$\begin{aligned} S(\mathrm{A|E}) &\geq \int d^2\alpha\ Q_{\checkmark}[\alpha]\cdot S_{\hat{\rho}^{\mathrm{f}}_{\mathrm{AE}}[\alpha]}(\mathrm{A|E}) \\ &= \int d^2\alpha\ Q_{\checkmark}[\alpha]\cdot\left[1-h_2\left(\frac{1+\left|\langle-\sqrt{r_{\mathrm{E}}}\alpha|\sqrt{r_{\mathrm{E}}}\alpha\rangle\right|}{2}\right)\right] \\ &= 1-\int d^2\alpha\ Q_{\checkmark}[\alpha]\cdot h_2\left(\frac{1+\exp\left(-2r_{\mathrm{E}}|\alpha|^2\right)}{2}\right), \end{aligned} \tag{121}$$

where $S_{\hat{\rho}^{\mathrm{f}}_{\mathrm{AE}}[\alpha]}(\mathrm{A|E})$ denotes conditional entropy for Alice-Eve density matrix described by Eq. (119). Utilizing Jensen's inequality,

$$\langle h_2(x)\rangle \leq h_2\left(\langle x\rangle\right), \tag{122}$$

we bound Eve's information as

$$I(\mathrm{A,E}) \leq h_2\left(\frac{1+\left\langle\exp\left(-2r_{\mathrm{E}}|\alpha|^2\right)\right\rangle_{Q_{\checkmark}}}{2}\right). \tag{123}$$

Straightforward calculations allows us to find

$$\begin{aligned} \left\langle\exp\left(-2r_{\mathrm{E}}|\alpha|^2\right)\right\rangle_{Q_{\checkmark}} &= \int d^2\alpha\ Q_{\checkmark}[\alpha]\cdot e^{-2r_{\mathrm{E}}|\alpha|^2} = \frac{\exp\left(\frac{-2r_{\mathrm{E}}|\gamma|^2}{1+2r_{\mathrm{E}}(G_1-1)}\right)}{2p_{\checkmark}\left(1+2r_{\mathrm{E}}(G_1-1)\right)} \\ &\times\left[\sum_{x=0,1}\mathrm{erf}\left(\frac{\theta_2'\cdot[1+2r_{\mathrm{E}}(G_1-1)]+(-1)^x\sqrt{(1-r_{\mathrm{E}})}\gamma}{\zeta\cdot\sqrt{1+2r_{\mathrm{E}}(G_1-1)}}\right)\right. \\ &\left.-\sum_{x=0,1}\mathrm{erf}\left(\frac{\theta_1'\cdot[1+2r_{\mathrm{E}}(G_1-1)]+(-1)^x\sqrt{(1-r_{\mathrm{E}})}\gamma}{\zeta\cdot\sqrt{1+2r_{\mathrm{E}}(G_1-1)}}\right)\right], \end{aligned} \tag{124}$$

where $\zeta = \sqrt{G_1+G_2+2r_{\mathrm{E}}(G_1-1)(G_2-1)-3/2}$. By substituting Eq. (124) into Eq. (123) we obtain the upper bound for Eve's information ($I(\mathrm{A{:}E})$ in the Eq. (6) of the main text).

References

79. Lesovik, G. B., Sadovskyy, I. A., Suslov, M. V., Lebedev, A. V. & Vinokur, V. M. Arrow of time and its reversal on the IBM quantum computer. *Sci. Rep.* **9**, 4396 (2019).
80. Kirsanov, N. S. *et al.* Entropy dynamics in the system of interacting qubits. *J.Russ. Laser Res.* **39**, 120–127 (2018).
81. Lesovik, G. B., Lebedev, A. V., Sadovskyy, I. A., Suslov, M. V. & Vinokur, V. M. H-theorem in quantum physics. *Sci. Rep.* **6**, 32815 (2016).
82. Holstein, T. & Primakoff, H. Field dependence of the intrinsic domain magnetization of a ferromagnet. *Phys. Rev.* **58**, 1098-1113 (1940).
83. Sanguinetti, B., Pomarico, E., Sekatski, P., Zbinden, H. & Gisin, N. Quantum cloning for absolute radiometry. *Phys. Rev. Lett.* **105**, 080503 (2010).
84. Vogel, W. & Welsch, D. *Quantum optics.* (John Wiley & Sons, 2006).
85. Sekatski, P., Sanguinetti, B., Pomarico, E., Gisin, N. & Simon, C. Cloning entangled photons to scales one can see. *Phys. Rev. A* **82**, 053814 (2010).
86. Zhao, Y., Qi, B. & Lo, H.-K. Experimental quantum key distribution with active phase randomization. *Appl. Phys. Lett.* **90**, 044106 (2007).
87. Abramowitz, M., Stegun, I. & Romer, R. *Handbook of mathematical functions with formulas, graphs, and mathematical tables* (American Association of Physics Teachers, 1988).
88. Lee Rodgers, J. & Nicewander, W. A. Thirteen ways to look at the correlation coefficient. *Am. Stat.* **42**, 59–66 (1988).
89. Pearson, K. VII. Mathematical contributions to the theory of evolution.—III. Regression, heredity, and panmixia. *Philos. T. R. Soc. Lond.* **187**, 253–318 (1896).
